# Synthetic data augmentation for CT-based emphysema subtype classification: A comparative evaluation of generative and classical approaches

**DOI:** 10.1371/journal.pone.0355850

**Published:** 2026-08-20

**Authors:** Nicholas Dietrich, David McShannon

**Affiliations:** 1 Temerty Faculty of Medicine, University of Toronto, Toronto, Ontario, Canada; 2 Faculty of Engineering, McMaster University, Hamilton, Ontario, Canada; Commonwealth Scientific and Industrial Research Organisation, AUSTRALIA

## Abstract

Data scarcity is a persistent challenge in medical image analysis. Synthetic data generation using deep generative models has been proposed as a potential approach to address this limitation, yet its performance in small-data settings remains poorly characterized. This study compared three class-conditional generative approaches, a conditional variational autoencoder (cVAE), a conditional shallow-decoder VAE variant (cSD-VAE), and a conditional Wasserstein GAN with gradient penalty (cWGAN-GP), against classical geometric augmentation for emphysema subtype classification on 168 CT patches (three classes: normal tissue, centrilobular emphysema, and paraseptal emphysema). Each method was evaluated at three synthetic-to-real ratios (0.5x, 1.0x, 2.0x) using patient-level 70/30 splits across 10 random seeds, with an ImageNet-pretrained ResNet18 as the downstream classifier. No individual augmentation strategy produced a statistically significant improvement in balanced accuracy over the unaugmented baseline (0.522 ± 0.067). The conditional WGAN-GP at 1.0x achieved the highest individual balanced accuracy (0.548 ± 0.068, Cohen’s d = 0.47 versus baseline), but did not reach statistical significance (p = 0.084). A pre-specified ensemble combining all four augmentation methods at the 1.0x multiplier did not significantly improve balanced accuracy over baseline (0.541 ± 0.088 versus 0.522 ± 0.067; Cohen’s d = 0.32, Wilcoxon p = 0.275). Neither pixel-space nor feature-space distributional fidelity was associated with downstream classification performance. Overall, no benefit was detected from class-conditional generative augmentation in this small-data, texture-driven setting. Future work should focus on improving generative modeling under small-data conditions, including task-aware objectives and pathology-constrained synthesis.

## Introduction

Chronic obstructive pulmonary disease (COPD) is a leading cause of morbidity and mortality worldwide, and emphysema, one of its principal components, is characterized by irreversible destruction of lung parenchyma [1]. Computed tomography (CT) allows for direct visualization of emphysematous changes, and texture-based analysis of CT image patches has become a widely used strategy for characterizing emphysema subtypes, including normal tissue (NT), centrilobular emphysema (CLE), and paraseptal emphysema (PSE) [2].

Deep learning methods have demonstrated strong performance across a wide range of medical imaging tasks, including image classification and pattern recognition [3–6]. However, these models typically require large, diverse training datasets to achieve robust generalization and avoid overfitting. In many medical imaging applications, including emphysema subtype classification, labeled datasets are limited in size, motivating the use of data augmentation strategies to improve model performance [7,8].

Classical data augmentation approaches apply geometric and photometric transformations (e.g., rotations, flips, scaling, and intensity perturbations) to existing images, generating modified samples that preserve diagnostic content while introducing controlled variability [9]. More recently, deep generative models, such as variational autoencoders (VAEs) and generative adversarial networks (GANs), have been proposed as an alternative strategy for data augmentation [10,11]. These approaches aim to synthesize new samples that approximate the underlying data distribution, with the goal of expanding the effective training set beyond simple transformations of existing data.

Prior work has demonstrated the potential of generative data augmentation across medical imaging domains [12–14]. However, generative models must themselves be trained on the available data, and in small-data settings may produce limited diversity or overfit to the training distribution. This has been examined in small-data medical imaging, where single-generation GAN-based augmentation did not consistently outperform classical augmentation and underperformed in smaller-data regimes [15]. However, empirical evaluation of generative augmentation methods on small medical imaging datasets remains limited.

This study addresses this gap by comparing generative and classical augmentation strategies for emphysema subtype classification in a small-data setting. The primary aim was to determine whether class-conditional VAE-based, GAN-based, or classical augmentation improves classification performance under limited data conditions. A secondary aim was to evaluate whether the distributional fidelity of synthetic data, as measured by maximum mean discrepancy (MMD), is associated with downstream classifier performance.

## Materials and methods

### Study design

This was a retrospective computational study evaluating synthetic data augmentation strategies for a three-class emphysema subtype classification task. This study was designed and reported in accordance with the Checklist for Artificial Intelligence in Medical Imaging (CLAIM) (S1 Checklist). The study used a publicly available benchmark dataset which did not involve new human data, or data that could identify individual patients during or after data collection [16]. All code was executed in a stable environment using a single NVIDIA A100 GPU.

### Dataset

The Computed Tomography Emphysema Database [16,17] was used, accessed on January 5, 2026. It comprises 168 patches of 61 x 61 pixels extracted from high-resolution CT slices of 39 de-identified patients (9 never-smokers, 10 healthy smokers, and 20 smokers with COPD). CT scanning was performed with a GE LightSpeed QX/i scanner using in-plane resolution of 0.78 x 0.78 mm, slice thickness of 1.25 mm, tube voltage of 140 kV, and tube current of 200 mAs, with reconstruction using a high-spatial-resolution (bone) algorithm. The 168 patches are distributed across three classes: 59 normal tissue (NT, from never-smokers), 50 centrilobular emphysema (CLE), and 59 paraseptal emphysema (PSE). Each patch is associated with a patient identifier to enable patient-level cross-validation. Patch labels were assigned by consensus of an experienced chest radiologist and a CT-experienced pulmonologist based on the leading pattern in the source slice.

### Data partitioning

For each of 10 experimental seeds, the 39 unique patients were randomly permuted and split into 70% training and 30% testing sets at the patient level, ensuring that all patches from a given patient appeared exclusively in either the training or the testing partition. This yielded training sets of 108–125 patches (mean 114.6) and test sets of 43–60 patches (mean 53.4) per seed. Class distributions in the test set varied across seeds due to the patient-level splitting and the unequal number of patches per patient. The 10 seeds were the integers 43–52. Subject permutations were generated with NumPy (version 2.0.2) using np.random.seed followed by np.random.permutation. Per-seed split composition and per-class training counts are provided in S1 Table.

### Classifier architecture

A ResNet18 [18] pretrained on ImageNet (IMAGENET1K_V1 weights) [18,19] was used as the downstream classifier. As the emphysema patches are single-channel 16-bit grayscale images, a learned 1-to-3 channel convolutional adapter (1x1 kernel, initialized to uniform weights) was prepended to the pretrained backbone. The final fully connected layer was replaced with a three-class output head. All parameters, including the pretrained backbone, were fine-tuned during training. The network was trained for 150 epochs using AdamW optimization (learning rate 0.001, weight decay 1e-4) with cross-entropy loss and a step learning rate scheduler (halving every 50 epochs, gamma = 0.5). Batch size was 16. Input patches were min-max normalized to [0, 1]. The same architecture and training protocol were applied uniformly across all experimental conditions.

### Generative models

**Fig 1:**
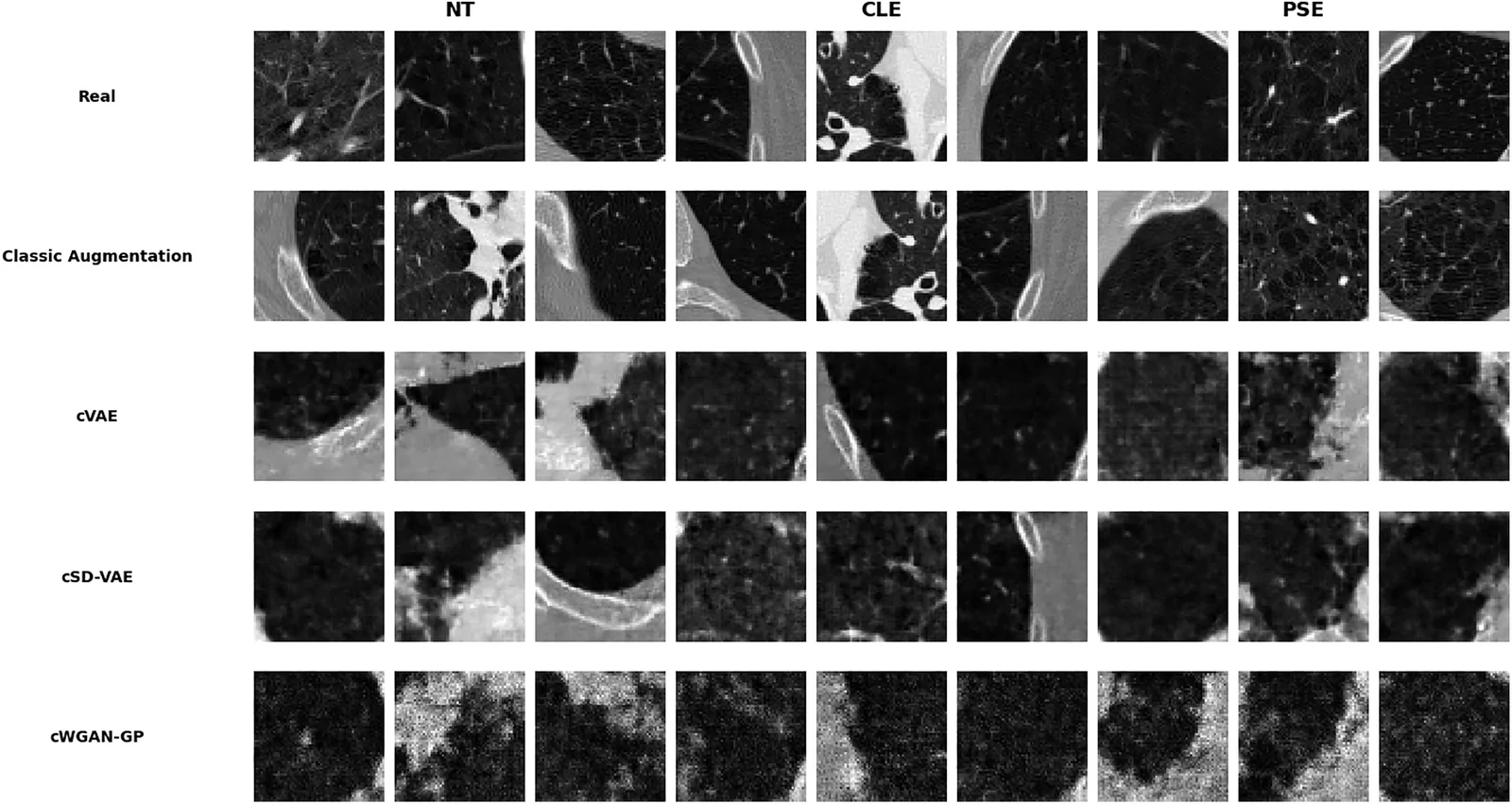
Representative computed tomography (CT) patches by emphysema subtype and augmentation method. Columns correspond to normal tissue (NT), centrilobular emphysema (CLE), and paraseptal emphysema (PSE). Rows show real images, classical augmentation, conditional variational autoencoder (cVAE), conditional shallow-decoder variational autoencoder (cSD-VAE), and conditional Wasserstein generative adversarial network with gradient penalty (cWGAN-GP).

Fig 1 shows representative examples of the CT patches for each class and synthetic augmentation strategy. Three class-conditional generative architectures were trained to produce synthetic emphysema patches. All generators received the class label as input, enabling per-class specialization of the learned latent representations. Each generative model was a single class-conditional network conditioned on the class label, so generation for a given class drew only on that class’s training patches.

The first was a conditional VAE (cVAE) with a convolutional encoder (four layers with 32, 64, 128, and 256 filters) whose output was concatenated with a 64-dimensional class embedding before projection to a 128-dimensional latent space. The decoder received the latent vector concatenated with the same class embedding. The cVAE was trained with mean squared error reconstruction loss plus a KL-divergence term (weight 1.0) using Adam optimization (learning rate 1e-3) for 1000 epochs.

The second was a conditional shallow-decoder VAE (cSD-VAE), identical in architecture to the cVAE but trained with a reduced KL weight (0.5) and lower learning rate (5e-4), intended to produce reconstructions closer to the input distribution at the cost of less regularized latent space.

The third was a conditional Wasserstein GAN with gradient penalty (cWGAN-GP). The generator received a 100-dimensional noise vector concatenated with a 64-dimensional class embedding, processed through a fully connected layer with batch normalization and four transposed-convolutional layers (256, 128, 64, 32 filters) to produce 61 x 61 single-channel images. The critic received the image concatenated with a spatial class map (produced by reshaping a learned 3,721-dimensional class embedding into a 61 x 61 additional input channel). Training used Adam optimization (learning rate 1e-4, betas 0.0 and 0.9) with 5 critic updates per generator update and a gradient penalty coefficient of 10.0, following the protocol of Gulrajani et al. [20]. Training ran for 1000 epochs.

All generative models were trained exclusively on the training-set patches for each seed. No test data were used for generator training.

### Classical augmentation

A geometric augmentation pipeline was implemented as the non-generative comparator. Each augmented sample was produced by randomly selecting a training patch, applying a random rotation (0, 90, 180, or 270 degrees), and optionally applying a horizontal flip (probability 0.5). This approach generates plausible variants that preserve the tissue texture while introducing spatial diversity.

### Augmentation protocol

For each generative method and for classical augmentation, synthetic samples were produced at three multipliers relative to the per-class training count: 0.5x, 1.0x, and 2.0x. The number of synthetic samples per class was proportional to the number of real training samples in that class, preserving the original class proportions. For cVAE and cSD-VAE, synthetic samples were generated by encoding a randomly selected same-class training patch with the corresponding class label, sampling from the latent space with reduced variance (standard deviation scaled to 0.5 of the learned distribution to reduce sampling noise), and decoding conditioned on the target class. For cWGAN-GP, samples were generated by passing random noise vectors along with the target class label through the generator. Synthetic samples were concatenated with the original training data and used together for classifier training. This yielded 13 experimental conditions: baseline (no augmentation), and four methods at three multipliers each. An additional ensemble condition was specified a priori, before examining any results, as the majority vote of all four augmentation methods at the 1.0x multiplier (cVAE, cSD-VAE, cWGAN-GP, and classical augmentation). As the ensemble members were fixed in advance, no test-set model selection was involved.

### Evaluation metrics

The primary outcome metric was balanced accuracy (the unweighted mean of per-class recall). Secondary metrics included overall accuracy, macro-averaged F1 score, weighted F1 score, macro-averaged area under the receiver operating characteristic curve (AUROC), and macro-averaged area under the precision-recall curve (AUPRC). Per-class sensitivity, specificity, precision, negative predictive value (NPV), and F1 score were computed from confusion matrices. All metrics were computed on the held-out test set for each seed.

### Distributional fidelity

Two complementary MMD measures were computed. Pixel-space MMD was computed directly on the flattened patch intensities. As classical augmentation reuses the original pixel intensities of real patches, its pixel-space MMD may be lower by construction, reflecting the transformation itself rather than serving as a fully independent measure of fidelity. To provide a measure less sensitive to this asymmetry, we additionally computed a feature-space MMD on the 512-dimensional activations of the penultimate ResNet18 layer (the global average-pooling output of the baseline classifier trained on the same seed), extracted for real and synthetic patches. For both measures, the statistic was computed over min(n_real, n_synth, 200) samples from each set, and the Gaussian kernel bandwidth was set by the median heuristic, taking the median of the pairwise squared Euclidean distances over up to 50 samples per set and setting the kernel coefficient to gamma = 1 / (2 x median) [21]. The feature-space MMD was computed independently within each seed, using that seed’s own baseline classifier as the encoder; feature-space values are therefore expressed in a seed-specific representation and are used only in within-seed association with balanced accuracy rather than compared in absolute terms across seeds. Lower MMD values indicate greater distributional similarity.

### Statistical analysis

Metrics were summarized as mean ± standard deviation across 10 seeds. The Friedman test was used to evaluate whether performance differed across all 14 conditions. Cohen’s d for paired samples was computed as the mean of the paired differences divided by their standard deviation. A post-hoc power analysis indicated that, with 10 seeds and the observed variability, the minimum detectable paired effect size at 80% power (two-sided alpha = 0.05) was Cohen’s d = 0.89. Spearman rank correlation was used to assess the association between MMD and balanced accuracy across all method-seed combinations. Pairwise comparisons against the unaugmented baseline used two-sided Wilcoxon signed-rank tests. To account for multiple comparisons, Bonferroni correction was applied across the 13 pairwise tests (12 individual augmentation conditions and the pre-specified ensemble), giving an adjusted significance threshold of alpha = 0.05/13 = 0.0038. Both uncorrected and Bonferroni-corrected p-values are reported as appropriate.

## Results

### Overall classification performance

Table 1 presents the primary performance metrics across all 14 experimental conditions. The baseline classifier (pretrained ResNet18, no augmentation) achieved a mean balanced accuracy of 0.522 ± 0.067 across 10 seeds. No individual augmentation strategy produced a statistically significant difference in balanced accuracy relative to baseline (Wilcoxon signed-rank test p-values > 0.05). Detailed per-seed results for all methods are provided in S2 Table.

**Table 1:** Classification performance across augmentation strategies (mean ± SD, 10 seeds).

Method	Balanced Accuracy	Accuracy	AUROC (macro)	AUPRC (macro)	F1 (macro)
**Baseline**	0.522 ± 0.067	0.467 ± 0.099	0.676 ± 0.085	0.536 ± 0.100	0.443 ± 0.084
**cVAE 0.5x**	0.516 ± 0.077	0.458 ± 0.114	0.664 ± 0.078	0.523 ± 0.082	0.439 ± 0.095
**cVAE 1.0x**	0.513 ± 0.063	0.437 ± 0.074	0.639 ± 0.075	0.485 ± 0.077	0.419 ± 0.068
**cVAE 2.0x**	0.509 ± 0.059	0.436 ± 0.074	0.643 ± 0.068	0.474 ± 0.070	0.418 ± 0.064
**cSD-VAE 0.5x**	0.524 ± 0.060	0.458 ± 0.097	0.664 ± 0.060	0.507 ± 0.067	0.437 ± 0.080
**cSD-VAE 1.0x**	0.503 ± 0.060	0.445 ± 0.074	0.641 ± 0.067	0.488 ± 0.076	0.435 ± 0.066
**cSD-VAE 2.0x**	0.508 ± 0.062	0.446 ± 0.072	0.655 ± 0.046	0.504 ± 0.048	0.436 ± 0.071
**cWGAN-GP 0.5x**	0.525 ± 0.069	0.458 ± 0.082	0.667 ± 0.061	0.510 ± 0.072	0.442 ± 0.079
**cWGAN-GP 1.0x**	0.548 ± 0.068	0.491 ± 0.089	0.675 ± 0.073	0.519 ± 0.081	0.472 ± 0.084
**cWGAN-GP 2.0x**	0.494 ± 0.089	0.425 ± 0.086	0.643 ± 0.092	0.493 ± 0.102	0.409 ± 0.088
**Classic Aug 0.5x**	0.528 ± 0.058	0.447 ± 0.074	0.671 ± 0.054	0.524 ± 0.058	0.430 ± 0.075
**Classic Aug 1.0x**	0.520 ± 0.071	0.440 ± 0.113	0.658 ± 0.062	0.498 ± 0.067	0.418 ± 0.098
**Classic Aug 2.0x**	0.523 ± 0.054	0.461 ± 0.078	0.660 ± 0.067	0.491 ± 0.068	0.445 ± 0.074
**Ensemble**	0.541 ± 0.088	0.468 ± 0.102	0.685 ± 0.079	0.524 ± 0.085	0.450 ± 0.091

cVAE: conditional variational autoencoder; cSD-VAE: conditional shallow-decoder variational autoencoder; cWGAN-GP: conditional Wasserstein generative adversarial network with gradient penalty; Classic Aug: classical augmentation; Ensemble: pre-specified majority-vote ensemble of all four augmentation methods at the 1.0x multiplier (cVAE, cSD-VAE, cWGAN-GP, classical augmentation). AUROC: area under the receiver operating characteristic curve; AUPRC: area under the precision–recall curve; F1: F1 score.

Among the generative methods, conditional WGAN-GP at 1.0x achieved the highest individual balanced accuracy (0.548 ± 0.068), exceeding baseline by 0.026 with a moderate effect size (Cohen’s d = 0.47), though the Wilcoxon p-value of 0.084 did not reach the pre-specified threshold. Performance for the conditional VAE methods tended to decline with increasing synthetic data volume (cVAE 0.5x: 0.516 ± 0.077; 1.0x: 0.513 ± 0.063; 2.0x: 0.509 ± 0.059), with a similar pattern for cSD-VAE (0.5x: 0.524 ± 0.060; 1.0x: 0.503 ± 0.060; 2.0x: 0.508 ± 0.062). The cWGAN-GP showed a non-monotonic pattern, with the best result at 1.0x and a notable decline at 2.0x (0.494 ± 0.089). Classical augmentation showed minimal variation across multipliers (0.5x: 0.528 ± 0.058; 1.0x: 0.520 ± 0.071; 2.0x: 0.523 ± 0.054), with none reaching significance versus baseline.

The pre-specified ensemble combining all four augmentation methods at the 1.0x multiplier did not significantly improve balanced accuracy over baseline (0.541 ± 0.088 versus 0.522 ± 0.067; Cohen’s d = 0.32, Wilcoxon p = 0.275). The Friedman test across the 14 conditions was not significant (chi-squared = 11.13, p = 0.600), indicating no overall difference in balanced accuracy across augmentation strategies.

**Fig 2:**
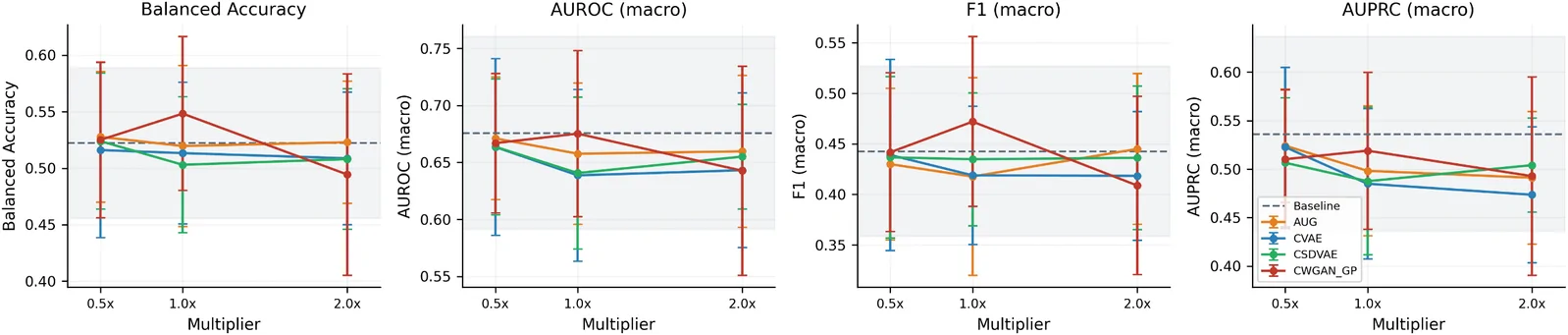
Effect of synthetic data volume on classification performance. Metrics shown include balanced accuracy, area under the receiver operating characteristic curve (AUROC; macro-average), F1 score (macro-average), and area under the precision–recall curve (AUPRC; macro-average). Error bars represent standard deviation across 10 seeds; the dashed line indicates the unaugmented baseline. In the legend, AUG denotes classical augmentation, CVAE the cVAE, CSDVAE the cSD-VAE, and CWGAN_GP the cWGAN-GP.

Macro-averaged AUROC showed a similar pattern. Baseline AUROC was 0.676 ± 0.085. The ensemble achieved an AUROC of 0.685 ± 0.079. The cWGAN-GP at 1.0x (0.675 ± 0.073) closely matched but did not exceed baseline. No individual augmentation condition exceeded baseline AUROC. At the uncorrected level, several generative conditions showed nominally significant deteriorations relative to baseline. The cVAE at 1.0x reduced AUPRC (raw p = 0.010) and AUROC (raw p = 0.027); the cSD-VAE at 1.0x reduced AUPRC (raw p = 0.014) and AUROC (raw p = 0.020); and the cVAE at 2.0x reduced AUPRC (raw p = 0.027). After Bonferroni correction for 13 comparisons, none of these deteriorations remained significant (all corrected p ≥ 0.13). All pairwise comparisons and corresponding effect sizes for all methods are provided in S3 Table. Fig 2 provides a summary of the effect of synthetic data volume on classification performance.

### Per-class performance

Class-level analysis revealed a consistent pattern across all conditions: PSE was the most readily classified subtype (baseline sensitivity 0.758 ± 0.179), NT had intermediate sensitivity (0.552 ± 0.176), and CLE was the most difficult class (baseline sensitivity 0.257 ± 0.141). This hierarchy persisted regardless of augmentation strategy.

For CLE, the most clinically relevant and poorly classified subtype, cWGAN-GP at 1.0x produced the largest improvement in mean sensitivity (0.385 ± 0.209 versus baseline 0.257 ± 0.141), with a corresponding CLE F1 of 0.401 ± 0.158. The cSD-VAE at 1.0x also showed a modest CLE sensitivity gain (0.362 ± 0.164). However, a Wilcoxon signed-rank test for CLE sensitivity was not significant for cWGAN-GP at 1.0x (raw p = 0.074; Bonferroni-corrected p = 0.96), and no augmentation method significantly improved CLE sensitivity (S4 Table). The a priori ensemble achieved a CLE sensitivity of 0.321 ± 0.195 with a CLE F1 of 0.342 ± 0.103. As an exploratory analysis prompted by the variation in per-seed CLE training counts under patient-level splitting (range 18–43; S1 Table), we also assessed whether this count was associated with augmentation benefit. The per-seed CLE training count was not significantly associated with the change in balanced accuracy relative to baseline (mean of the four methods at the 1.0x multiplier; Spearman rho = −0.29, p = 0.41).

PSE sensitivity was highest at baseline (0.758 ± 0.179) and tended to decrease modestly with augmentation, particularly with generative methods at higher multipliers (for example, cSD-VAE 2.0x: 0.631 ± 0.207, cWGAN-GP 2.0x: 0.675 ± 0.240). Per-class performance metrics across all methods and seeds are provided in S5 Table and corresponding confusion matrices for each method and seed are provided in S6 Table.

### Distributional fidelity

Pixel-space MMD values are presented in Table 2. Classical augmentation produced among the lowest MMD values (0.5x: 0.009 ± 0.003; 1.0x: 0.006 ± 0.002; 2.0x: 0.006 ± 0.002). cWGAN-GP achieved comparably low MMD values (0.5x: 0.010 ± 0.002; 1.0x: 0.006 ± 0.002; 2.0x: 0.007 ± 0.003), indicating that the gradient penalty and class-conditional architecture enabled the GAN to produce samples distributionally close to the real training data. The conditional VAE methods showed intermediate MMD values (range 0.020 to 0.030).

**Table 2:** Distributional fidelity (pixel-space MMD, mean ± SD) and synthetic sample counts across 10 seeds.

Method	MMD	N synthetic
cVAE 0.5x	0.028 ± 0.008	57
cVAE 1.0x	0.026 ± 0.005	114
cVAE 2.0x	0.020 ± 0.005	229
cSD-VAE 0.5x	0.030 ± 0.011	57
cSD-VAE 1.0x	0.027 ± 0.007	114
cSD-VAE 2.0x	0.020 ± 0.006	229
cWGAN-GP 0.5x	0.010 ± 0.002	57
cWGAN-GP 1.0x	0.006 ± 0.002	114
cWGAN-GP 2.0x	0.007 ± 0.003	229
Classic Aug 0.5x	0.009 ± 0.003	57
Classic Aug 1.0x	0.006 ± 0.002	114
Classic Aug 2.0x	0.006 ± 0.002	229

MMD: maximum mean discrepancy. cVAE: conditional variational autoencoder; cSD-VAE: conditional shallow-decoder variational autoencoder; cWGAN-GP: conditional Wasserstein generative adversarial network with gradient penalty; Classic Aug: classical augmentation. N synthetic: mean number of synthetic samples generated per condition.

**Fig 3:**
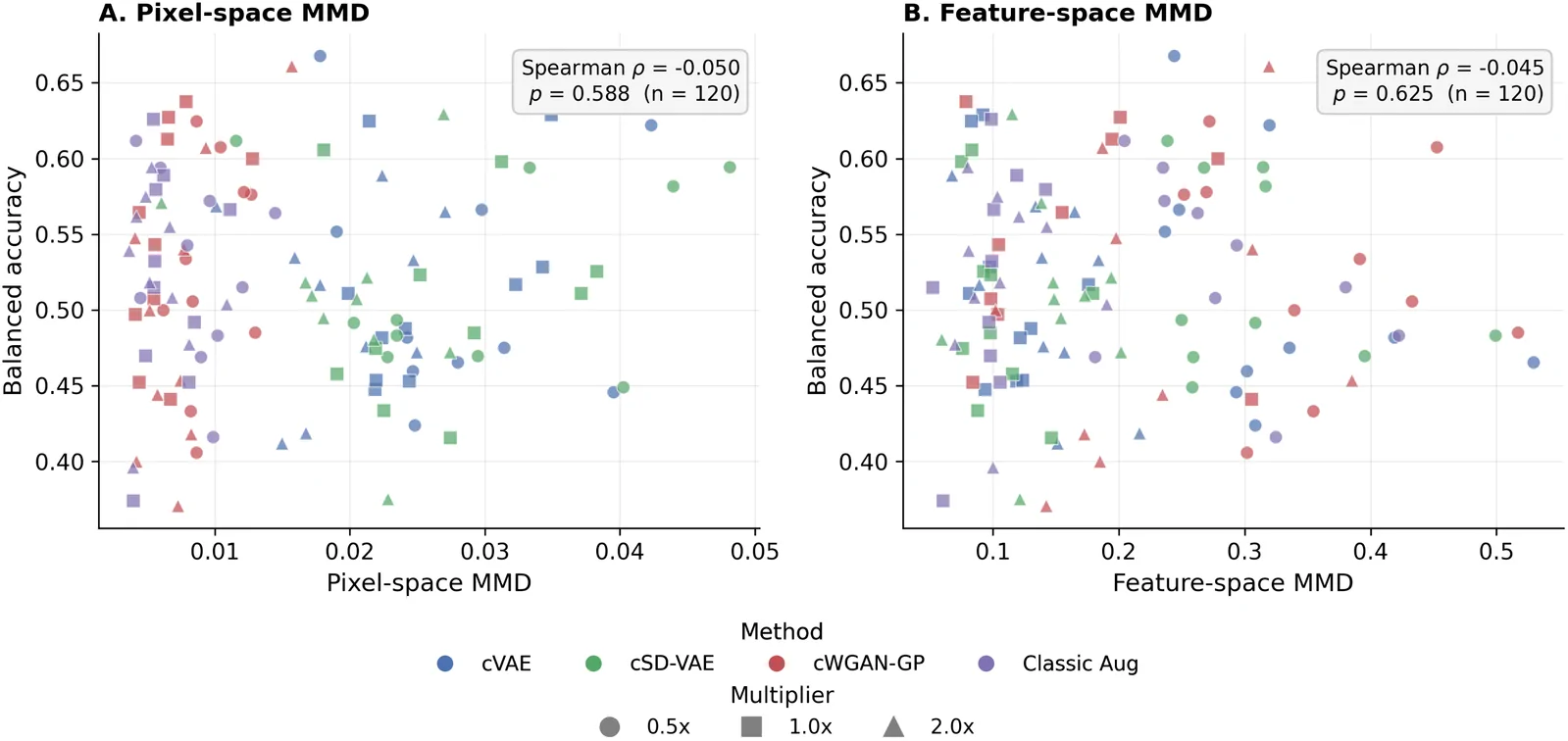
Distributional fidelity versus classification performance. (A) Pixel-space maximum mean discrepancy (MMD) and (B) feature-space MMD (penultimate ResNet18 activations), each plotted against balanced accuracy across all 120 method-seed observations (12 augmentation conditions x 10 seeds). Spearman rank correlation coefficients are shown in each panel. Aug: classical augmentation; cVAE: conditional variational autoencoder; cSD-VAE: conditional shallow-decoder variational autoencoder; cWGAN-GP: conditional Wasserstein generative adversarial network with gradient penalty.

Feature-space MMD did not follow the same ordering across methods as pixel-space MMD; in particular, cWGAN-GP, which produced among the lowest pixel-space MMD values, did not rank lowest in feature space. Full per-seed pixel and feature MMD values are provided in S7 Table. The Spearman rank correlation between MMD and balanced accuracy across all 120 method-seed observations was negligible in both pixel space (rho = −0.050, p = 0.588) and feature space (rho = −0.045, p = 0.625), indicating no association between distributional fidelity and downstream classification performance at either level (Fig 3). As the feature-space MMD pools observations from seed-specific embeddings, this correlation should be interpreted as exploratory.

## Discussion

This study evaluated whether synthetic data augmentation using three class-conditional generative approaches or classical geometric transformations improves emphysema subtype classification when training data is limited. Our principal finding was that no individual augmentation strategy produced a statistically significant improvement in balanced accuracy over the unaugmented baseline. These findings are relevant to clinical imaging research where labeled datasets are often limited and texture-based distinctions are critical, such as interstitial lung disease or emphysema phenotyping.

The baseline classifier achieved a mean balanced accuracy of 0.522, which is above chance (0.333 for three classes) but reflects the inherent difficulty of the task on this small, imbalanced dataset. The use of an ImageNet-pretrained ResNet18 was intended to provide a stronger baseline through transfer learning. In practice, the pretrained features did not produce a marked advantage for this task, likely because the ImageNet domain (RGB natural images) differs substantially from single-channel 16-bit CT textures. This is consistent with findings in the transfer learning literature showing that domain distance between source and target tasks modulates the benefit of pretraining [22,23].

The nominal AUROC and AUPRC deteriorations observed for the conditional VAE variants did not survive multiple-comparison correction, but they are consistent with the absence of any benefit from VAE-based augmentation in this setting. For the VAE models, latent sampling variance was reduced by scaling the standard deviation to 0.5 to limit noisy or implausible outputs. However, this may also have reduced sample diversity and limited the value of the generated images for augmentation. This offers one possible explanation for why the VAEs did not outperform classical augmentation and motivates further work on task-aware sampling. In addition, the pre-specified ensemble combining all four augmentation methods at 1.0x did not significantly improve balanced accuracy over baseline (0.541 ± 0.088; Cohen’s d = 0.32; p = 0.275). While ensemble methods can improve generalization by aggregating diverse, partially uncorrelated models [24,25], the constituent augmentation methods here did not individually outperform baseline, so combining them did not add detectable discriminative signal. This is consistent with the absence of a benefit from any single method. It is important to note that other pre-specified ensemble definitions, or a validation-based selection using a three-way split within each seed, might yield different results; we fixed a single transparent rule (all methods at the 1.0x multiplier) in advance to avoid selection on the test set.

Furthermore, the correlation between MMD and downstream performance was negligible in both pixel space (rho = −0.050, p = 0.588) and feature space (rho = −0.045, p = 0.625). The absence of an association even in feature space suggests that this is not merely an artifact of measuring fidelity in pixel space; aggregate distributional similarity, however measured, did not predict task performance in this setting. Samples that are distributionally similar to real data may nevertheless fail to introduce informative variation along the discriminative dimensions required for classification. In addition, the feature-space measure was derived from the baseline-trained encoder and may therefore under-represent structure that an encoder not optimized on the baseline task would capture. Nonetheless, the consistent lack of association in both pixel and feature space supports the overall finding, although this should not be interpreted as evidence that no association exists.

CLE was consistently the most difficult class to classify, with baseline sensitivity of 0.257 ± 0.141. cWGAN-GP at 1.0x showed the highest CLE sensitivity among individual methods (0.385 ± 0.209), but this apparent improvement was not statistically significant (raw p = 0.074; corrected p = 0.96) and was accompanied by substantial cross-seed variance. This difficulty aligns with prior literature on the Sørensen et al. database [16], where CLE patches exhibit more heterogeneous and subtle textural patterns compared to the more distinctive appearances of NT and PSE [26,27]. The trade-off between CLE sensitivity and PSE sensitivity observed across conditions suggests that augmentation strategies may redistribute classifier sensitivity among classes without expanding overall discriminative capacity.

Most studies using the emphysema patch database have employed hand-crafted feature descriptors, such as local binary patterns, Gabor filters, and co-occurrence matrices, with classical machine learning classifiers achieving reported accuracies in the range of 0.70 to 0.92 on various cross-validation schemes [16, 26–28]. The lower performance observed in the present study likely reflects the use of patient-level splitting, which is stricter than the evaluation in some of the cited works [16,26], combined with a classification task focused on raw patch inputs rather than engineered features. This design choice was intentional, as the goal was to isolate the effect of data augmentation rather than to achieve state-of-the-art classification.

Recent medical imaging literature also indicates that generative augmentation is not uniformly beneficial [12,13,29]. While classical affine transformations remain the most consistently effective and widely adopted strategy, the performance of generative methods varies substantially depending on dataset size, imaging modality, and task complexity [7,9,14]. Consistent with prior work [29–31], our findings show that even under methodologically rigorous conditions, generative augmentation does not reliably improve classification performance in small-data settings.

However, these findings should be contextualized by our exclusion of diffusion-based generative models. Although diffusion models have historically been regarded as data-intensive, recent work has shown that pretrained and fine-tuned diffusion models can produce useful augmentation in small-data medical imaging. For example, adapting a large pretrained generative model with lightweight per-class adapters improved downstream classification on medical datasets of a few thousand images [32], and diffusion-based synthesis has been applied as an augmentation strategy on small-volume medical benchmarks [33]. As the present study evaluates only VAE- and GAN-family models, our conclusions are limited to those families, and a pretrained-and-fine-tuned diffusion comparator is a relevant direction for future work.

Several limitations should be acknowledged. First, the dataset contains only 168 patches from 39 patients, which limits both the training capacity of generative models and the statistical power of comparative analyses. Second, the generative architectures employed were relatively compact, as more complex models (e.g., diffusion models or StyleGAN variants) have typically required substantially larger training datasets and computational resources to train effectively [30,34,35]. Whether higher-capacity generative models would alter these findings remains uncertain, particularly given the fundamental constraint of limited source data diversity.

In addition, the generative comparators were not individually tuned. The cVAE and cSD-VAE used a single, untuned configuration, and the cSD-VAE differed from the cVAE in both KL weight (0.5 versus 1.0) and learning rate (5e-4 versus 1e-3); consequently, differences between the two VAE variants cannot be attributed to any single hyperparameter. The cWGAN-GP used the canonical settings of Gulrajani et al. without further tuning [20]. The reported VAE results therefore reflect a single configuration rather than an optimized one, and a more extensive hyperparameter search could change the VAE performance.

We did not assess optimization dynamics, such as gradient norms or loss-landscape curvature, and therefore could not determine whether the decline at the 2.0x multiplier reflected synthetic-data dominance or training instability. We also did not perform an ablation comparing the ImageNet-pretrained classifier with a randomly initialized one, so the contribution of transfer learning in this domain-mismatched setting was not directly quantified. The ResNet18 backbone was not specifically optimized for single-channel CT texture, and our conclusions are conditional on this choice. Lastly, the study did not evaluate augmentation strategies in combination, such as applying geometric transformations to generatively augmented data, which could yield different outcomes.

## Conclusions

In a controlled comparison on a small emphysema CT patch dataset, generative and classical geometric augmentation did not produce statistically significant improvements in emphysema subtype classification when applied individually or with a pre-specified ensemble combining all four augmentation methods. These findings suggest there may be limited standalone utility of generative augmentation in small-data, texture-driven tasks. Future work should focus on improving generation under small-data conditions using task-aware and pathology-constrained strategies.

## Supporting information

S1 Checklist Completed Checklist for Artificial Intelligence in Medical Imaging (CLAIM). (DOCX)

S1 Table Per-seed per-class training-set patch counts. NT: normal tissue; CLE: centrilobular emphysema; PSE: paraseptal emphysema. Seeds are the integers 43–52.
(DOCX)

S2 Table Per-seed, per-method values for all classification metrics. AUROC: area under the receiver operating characteristic curve (macro-average); AUPRC: area under the precision–recall curve (macro-average); F1 (macro): macro-averaged F1 score; F1 (weighted): weighted F1 score; MMD: pixel-space maximum mean discrepancy. cVAE: conditional variational autoencoder; cSD-VAE: conditional shallow-decoder variational autoencoder; cWGAN-GP: conditional Wasserstein generative adversarial network with gradient penalty; aug: classical augmentation; ensemble: pre-specified majority-vote ensemble of all four augmentation methods at the 1.0x multiplier. N Train: number of training samples; N Synth: number of synthetic samples.
(DOCX)

S3 Table Wilcoxon signed-rank test results for all pairwise comparisons against baseline. AUROC: area under the receiver operating characteristic curve (macro-average); AUPRC: area under the precision–recall curve (macro-average); F1 (macro): macro-averaged F1 score. cVAE: conditional variational autoencoder; cSD-VAE: conditional shallow-decoder variational autoencoder; cWGAN-GP: conditional Wasserstein generative adversarial network with gradient penalty; aug: classical augmentation; ensemble: pre-specified majority-vote ensemble of all four augmentation methods at the 1.0x multiplier.
(DOCX)

S4 Table Per-class CLE sensitivity for each augmentation method versus baseline, with mean, standard deviation, paired difference, Cohen’s d, and uncorrected and Bonferroni-corrected Wilcoxon p-values. CLE: centrilobular emphysema. ensemble: pre-specified a priori ensemble (all methods at 1.0x).
(DOCX)

S5 Table Per-seed, per-method, per-class sensitivity, specificity, precision, NPV, and F1. NPV: negative predictive value; F1: F1 score. cVAE: conditional variational autoencoder; cSD-VAE: conditional shallow-decoder variational autoencoder; cWGAN-GP: conditional Wasserstein generative adversarial network with gradient penalty; aug: classical augmentation; ensemble: pre-specified majority-vote ensemble of all four augmentation methods at the 1.0x multiplier. CLE: centrilobular emphysema; PSE: paraseptal emphysema.
(DOCX)

S6 Table Per-seed confusion matrices for all methods. NT: normal tissue; CLE: centrilobular emphysema; PSE: paraseptal emphysema. cVAE: conditional variational autoencoder; cSD-VAE: conditional shallow-decoder variational autoencoder; cWGAN-GP: conditional Wasserstein generative adversarial network with gradient penalty; aug: classical augmentation; ensemble: pre-specified majority-vote ensemble of all four augmentation methods at the 1.0x multiplier. “True” and “Pred” denote ground-truth and predicted class labels, respectively.
(DOCX)

S7 Table Per-seed pixel-space and feature-space maximum mean discrepancy (MMD) for each augmentation method, alongside the corresponding balanced accuracy. Feature-space MMD was computed on the 512-dimensional penultimate ResNet18 activations. cVAE: conditional variational autoencoder; cSD-VAE: conditional shallow-decoder variational autoencoder; cWGAN-GP: conditional Wasserstein generative adversarial network with gradient penalty; aug: classical augmentation.
(DOCX)
