# Supplementary material for: Synthetic data augmentation for CT-based emphysema subtype classification: A comparative evaluation of generative and classical approaches
Source: PLoS One. 2026 Aug 20;21(8):e0355850. doi: 10.1371/journal.pone.0355850 (PMC13492795; doi:10.1371/journal.pone.0355850)
Supplement: Supplementary file 1 [file pone.0355850.s001.docx]

**Checklist for Artificial Intelligence in Medical Imaging (CLAIM): 2024 Update**

| Section / Topic | No. | Item | Page / Line | No | NA |
| --- | --- | --- | --- | --- | --- |
| TITLE / ABSTRACT |  |  |  |  |  |
|  | **1** | Identification as a study of AI methodology, specifying the category of technology used (e.g., deep learning) | **Title page** |  |  |
| ABSTRACT |  |  |  |  |  |
|  | **2** | Summary of study design, methods, results, and conclusions | **Abstract page** |  |  |
| INTRODUCTION |  |  |  |  |  |
|  | **3** | Scientific and/or clinical background, including the intended use and role of the AI approach | **Y** |  |  |
|  | **4** | Study aims, objectives, and hypotheses | **Y** |  |  |
| METHODS |  |  |  |  |  |
| *Study Design* | **5** | Prospective or retrospective study | **Y** |  |  |
|  | **6** | Study goal | **Y** |  |  |
| *Data* | **7** | Data sources | **Y** |  |  |
|  | **8** | Inclusion and exclusion criteria |  |  | **X** |
|  | **9** | Data pre-processing | **Y** |  |  |
|  | **10** | Selection of data subsets | **Y** |  |  |
|  | **11** | De-identification methods |  |  | **X** |
|  | **12** | How missing data were handled |  |  | **X** |
|  | **13** | Image acquisition protocol | **Y** |  |  |
| *Reference Standard* | **14** | Definition of method(s) used to obtain reference standard | **Y** |  |  |
|  | **15** | Rationale for choosing the reference standard |  |  | **X** |
|  | **16** | Source of reference standard annotations | **Y** |  |  |
|  | **17** | Annotation of test set | **Y** |  |  |
|  | **18** | Measures of inter- and intra-rater variability of features described by the annotators |  |  | **X** |
| *Data Partitions* | **19** | How data were assigned to partitions | **Y** |  |  |
|  | **20** | Level at which partitions are disjoint | **Y** |  |  |
| *Testing Data* | **21** | Intended sample size | **Y** |  |  |

| Section / Topic | No. | Item | Page / Line | No | NA |
| --- | --- | --- | --- | --- | --- |
| *Model* | **22** | Detailed description of model | **Y** |  |  |
|  | **23** | Software libraries, frameworks, and packages | **Y** |  |  |
|  | **24** | Initialization of model parameters | **Y** |  |  |
| *Training* | **25** | Details of training approach | **Y** |  |  |
|  | **26** | Method of selecting the final model | **Y** |  |  |
|  | **27** | Ensembling techniques | **Y** |  |  |
| *Evaluation* | **28** | Metrics of model performance | **Y** |  |  |
|  | **29** | Statistical measures of significance and uncertainty | **Y** |  |  |
|  | **30** | Robustness or sensitivity analysis |  |  | **X** |
|  | **31** | Methods for explainability or interpretability |  |  | **X** |
|  | **32** | Evaluation on internal data | **Y** |  |  |
|  | **33** | Testing on external data |  |  | **X** |
|  | **34** | Clinical trial registration |  |  | **X** |
| RESULTS |  |  |  |  |  |
| *Data* | **35** | Numbers of patients or examinations included and excluded | **Y** |  |  |
|  | **36** | Demographic and clinical characteristics of cases in each partition |  |  | **X** |
| *Model performance* | **37** | Performance metrics and measures of statistical uncertainty | **Y** |  |  |
|  | **38** | Estimates of diagnostic performance and their precision | **Y** |  |  |
|  | **39** | Failure analysis of incorrect results |  |  | **X** |
| DISCUSSION |  |  |  |  |  |
|  | **40** | Study limitations | **Y** |  |  |
|  | **41** | Implications for practice, including intended use and/or clinical role | **Y** |  |  |
| OTHER INFORMATION |  |  |  |  |  |
|  | **42** | Provide a reference to the full study protocol or to additional technical details |  |  | **X** |
|  | **43** | Statement about the availability of software, trained model, and/or data | **Y** |  |  |
|  | **44** | Sources of funding and other support; role of funders | **Y** |  |  |

* Indicate page and/or line number for each checklist item that is present. NA = not applicable.
