# Supplementary material for: Synthetic data augmentation for CT-based emphysema subtype classification: A comparative evaluation of generative and classical approaches
Source: PLoS One. 2026 Aug 20;21(8):e0355850. doi: 10.1371/journal.pone.0355850 (PMC13492795; doi:10.1371/journal.pone.0355850)
Supplement: Supplementary file 2 [file pone.0355850.s002.docx]

**S1 Table.**

| **Seed** | **Train NT** | **Train CLE** | **Train PSE** |
| --- | --- | --- | --- |
| **43** | 43 | 21 | 44 |
| **44** | 31 | 43 | 36 |
| **45** | 38 | 18 | 52 |
| **46** | 43 | 24 | 50 |
| **47** | 49 | 28 | 48 |
| **48** | 44 | 29 | 49 |
| **49** | 49 | 38 | 29 |
| **50** | 41 | 19 | 49 |
| **51** | 33 | 28 | 54 |
| **52** | 48 | 31 | 37 |
