# Supplementary material for: Synthetic data augmentation for CT-based emphysema subtype classification: A comparative evaluation of generative and classical approaches
Source: PLoS One. 2026 Aug 20;21(8):e0355850. doi: 10.1371/journal.pone.0355850 (PMC13492795; doi:10.1371/journal.pone.0355850)
Supplement: Supplementary file 3 [file pone.0355850.s003.docx]

**S2 Table.**

| **Seed** | **Method** | **Accuracy** | **Balanced**  **Accuracy** | **F1**  **Macro** | **F1**  **Weighted** | **AUROC**  **Macro** | **AUPRC**  **Macro** | **N Train** | **N**  **Synth** | **MMD** |
| --- | --- | --- | --- | --- | --- | --- | --- | --- | --- | --- |
| **43** | baseline | 0.4833 | 0.5914 | 0.4732 | 0.4053 | 0.7597 | 0.6473 | 108 | 0 | 0 |
|  | cvae_0.5x | 0.4 | 0.4751 | 0.403 | 0.3577 | 0.712 | 0.5862 | 162 | 54 | 0.0314 |
|  | cvae_1.0x | 0.45 | 0.511 | 0.4566 | 0.428 | 0.7004 | 0.5617 | 216 | 108 | 0.0199 |
|  | cvae_2.0x | 0.4 | 0.5167 | 0.3684 | 0.2866 | 0.6091 | 0.4113 | 324 | 216 | 0.0178 |
|  | csdvae_0.5x | 0.45 | 0.5819 | 0.4 | 0.3096 | 0.7402 | 0.566 | 162 | 54 | 0.0439 |
|  | csdvae_1.0x | 0.5333 | 0.5979 | 0.5473 | 0.5151 | 0.7377 | 0.6189 | 216 | 108 | 0.0312 |
|  | csdvae_2.0x | 0.55 | 0.6295 | 0.5543 | 0.5158 | 0.7336 | 0.5686 | 324 | 216 | 0.0269 |
|  | cwgan_gp_0.5x | 0.5 | 0.5763 | 0.5121 | 0.4719 | 0.7005 | 0.6197 | 162 | 54 | 0.0127 |
|  | cwgan_gp_1.0x | 0.6167 | 0.6273 | 0.621 | 0.621 | 0.7642 | 0.6453 | 216 | 108 | 0.0066 |
|  | cwgan_gp_2.0x | 0.45 | 0.5404 | 0.4425 | 0.3922 | 0.6811 | 0.5165 | 324 | 216 | 0.0077 |
|  | aug_0.5x | 0.4667 | 0.564 | 0.4678 | 0.4064 | 0.7126 | 0.6225 | 162 | 54 | 0.0145 |
|  | aug_1.0x | 0.5 | 0.6258 | 0.4679 | 0.39 | 0.7302 | 0.5572 | 216 | 108 | 0.0055 |
|  | aug_2.0x | 0.5 | 0.5749 | 0.5071 | 0.4668 | 0.742 | 0.5574 | 324 | 216 | 0.0049 |
|  | ensemble | 0.5000 | 0.5670 | 0.5180 | 0.4829 | 0.7892 | 0.6480 | NA | NA | NA |
| **44** | baseline | 0.6379 | 0.5916 | 0.5725 | 0.6646 | 0.7816 | 0.6755 | 110 | 0 | 0 |
|  | cvae_0.5x | 0.6379 | 0.5663 | 0.5451 | 0.6372 | 0.7088 | 0.5678 | 166 | 56 | 0.0298 |
|  | cvae_1.0x | 0.5517 | 0.6289 | 0.5394 | 0.5935 | 0.7485 | 0.5893 | 220 | 110 | 0.0349 |
|  | cvae_2.0x | 0.5862 | 0.589 | 0.5458 | 0.6058 | 0.7612 | 0.573 | 330 | 220 | 0.0224 |
|  | csdvae_0.5x | 0.6379 | 0.5942 | 0.5737 | 0.6468 | 0.7167 | 0.5723 | 166 | 56 | 0.0333 |
|  | csdvae_1.0x | 0.5345 | 0.5254 | 0.4818 | 0.5473 | 0.6868 | 0.5504 | 220 | 110 | 0.0383 |
|  | csdvae_2.0x | 0.4483 | 0.4808 | 0.4416 | 0.5016 | 0.6798 | 0.5485 | 330 | 220 | 0.0218 |
|  | cwgan_gp_0.5x | 0.5172 | 0.5057 | 0.4744 | 0.5448 | 0.7072 | 0.5234 | 166 | 56 | 0.0084 |
|  | cwgan_gp_1.0x | 0.6207 | 0.6128 | 0.5752 | 0.6458 | 0.7685 | 0.6163 | 220 | 110 | 0.0065 |
|  | cwgan_gp_2.0x | 0.431 | 0.4001 | 0.4132 | 0.5054 | 0.6564 | 0.5487 | 330 | 220 | 0.0042 |
|  | aug_0.5x | 0.5345 | 0.5942 | 0.5002 | 0.5283 | 0.7286 | 0.5676 | 166 | 56 | 0.0059 |
|  | aug_1.0x | 0.6379 | 0.5663 | 0.5465 | 0.6482 | 0.6781 | 0.5522 | 220 | 110 | 0.0111 |
|  | aug_2.0x | 0.569 | 0.5083 | 0.4967 | 0.5906 | 0.6568 | 0.516 | 330 | 220 | 0.0068 |
|  | ensemble | 0.6724 | 0.6946 | 0.6281 | 0.6884 | 0.7793 | 0.6047 | NA | NA | NA |
| **45** | baseline | 0.35 | 0.5119 | 0.3523 | 0.3623 | 0.6725 | 0.4992 | 108 | 0 | 0 |
|  | cvae_0.5x | 0.2667 | 0.4598 | 0.2691 | 0.2448 | 0.5881 | 0.4564 | 162 | 54 | 0.0247 |
|  | cvae_1.0x | 0.35 | 0.5283 | 0.3472 | 0.3312 | 0.6313 | 0.4724 | 216 | 108 | 0.0343 |
|  | cvae_2.0x | 0.4 | 0.565 | 0.4054 | 0.4037 | 0.674 | 0.4911 | 324 | 216 | 0.027 |
|  | csdvae_0.5x | 0.3 | 0.4916 | 0.2981 | 0.2702 | 0.6566 | 0.5008 | 162 | 54 | 0.0203 |
|  | csdvae_1.0x | 0.3333 | 0.5233 | 0.3333 | 0.3004 | 0.6336 | 0.4622 | 216 | 108 | 0.0252 |
|  | csdvae_2.0x | 0.3167 | 0.5074 | 0.3165 | 0.2957 | 0.6427 | 0.4877 | 324 | 216 | 0.0205 |
|  | cwgan_gp_0.5x | 0.35 | 0.5337 | 0.3489 | 0.3242 | 0.6835 | 0.5219 | 162 | 54 | 0.0079 |
|  | cwgan_gp_1.0x | 0.3167 | 0.5074 | 0.3149 | 0.2902 | 0.6763 | 0.5162 | 216 | 108 | 0.0055 |
|  | cwgan_gp_2.0x | 0.2333 | 0.4444 | 0.2149 | 0.159 | 0.6475 | 0.4455 | 324 | 216 | 0.0058 |
|  | aug_0.5x | 0.3 | 0.5079 | 0.2807 | 0.2205 | 0.6889 | 0.5189 | 162 | 54 | 0.0045 |
|  | aug_1.0x | 0.2833 | 0.4921 | 0.26 | 0.1971 | 0.6792 | 0.5071 | 216 | 108 | 0.0085 |
|  | aug_2.0x | 0.3667 | 0.5551 | 0.3541 | 0.3132 | 0.6946 | 0.4851 | 324 | 216 | 0.0066 |
|  | ensemble | 0.3167 | 0.5074 | 0.3126 | 0.2820 | 0.6487 | 0.4809 | NA | NA | NA |
| **46** | baseline | 0.3529 | 0.4078 | 0.3416 | 0.3224 | 0.5474 | 0.3937 | 117 | 0 | 0 |
|  | cvae_0.5x | 0.3529 | 0.424 | 0.3491 | 0.3243 | 0.5583 | 0.4058 | 176 | 59 | 0.0248 |
|  | cvae_1.0x | 0.3725 | 0.453 | 0.3741 | 0.3516 | 0.5859 | 0.4148 | 234 | 117 | 0.0244 |
|  | cvae_2.0x | 0.3529 | 0.4724 | 0.3427 | 0.2878 | 0.577 | 0.4036 | 351 | 234 | 0.0249 |
|  | csdvae_0.5x | 0.3725 | 0.4692 | 0.3764 | 0.3479 | 0.5791 | 0.4255 | 176 | 59 | 0.0228 |
|  | csdvae_1.0x | 0.3922 | 0.4336 | 0.3937 | 0.3903 | 0.5695 | 0.4046 | 234 | 117 | 0.0225 |
|  | csdvae_2.0x | 0.3529 | 0.3755 | 0.3412 | 0.3457 | 0.5541 | 0.4005 | 351 | 234 | 0.0228 |
|  | cwgan_gp_0.5x | 0.4118 | 0.406 | 0.3919 | 0.4168 | 0.5495 | 0.3862 | 176 | 59 | 0.0086 |
|  | cwgan_gp_1.0x | 0.4118 | 0.5431 | 0.3997 | 0.346 | 0.5497 | 0.3988 | 234 | 117 | 0.0055 |
|  | cwgan_gp_2.0x | 0.3333 | 0.3709 | 0.3339 | 0.3277 | 0.5174 | 0.3646 | 351 | 234 | 0.0073 |
|  | aug_0.5x | 0.3725 | 0.469 | 0.3602 | 0.3238 | 0.5891 | 0.4502 | 176 | 59 | 0.009 |
|  | aug_1.0x | 0.3137 | 0.3741 | 0.3004 | 0.2785 | 0.5448 | 0.3925 | 234 | 117 | 0.004 |
|  | aug_2.0x | 0.3725 | 0.3964 | 0.3562 | 0.3642 | 0.5368 | 0.3597 | 351 | 234 | 0.0039 |
|  | ensemble | 0.3725 | 0.4368 | 0.3681 | 0.3535 | 0.5594 | 0.3837 | NA | NA | NA |
| **47** | baseline | 0.3953 | 0.4515 | 0.392 | 0.3724 | 0.6117 | 0.4633 | 125 | 0 | 0 |
|  | cvae_0.5x | 0.4419 | 0.4818 | 0.4373 | 0.4316 | 0.5885 | 0.4311 | 187 | 62 | 0.0242 |
|  | cvae_1.0x | 0.4186 | 0.4879 | 0.4219 | 0.3994 | 0.5891 | 0.4147 | 250 | 125 | 0.0241 |
|  | cvae_2.0x | 0.3721 | 0.4121 | 0.3768 | 0.3658 | 0.5527 | 0.4097 | 375 | 250 | 0.015 |
|  | csdvae_0.5x | 0.4419 | 0.4697 | 0.4425 | 0.4427 | 0.6125 | 0.4481 | 187 | 62 | 0.0295 |
|  | csdvae_1.0x | 0.4419 | 0.4848 | 0.4414 | 0.438 | 0.5687 | 0.3961 | 250 | 125 | 0.0292 |
|  | csdvae_2.0x | 0.4884 | 0.5182 | 0.4853 | 0.4928 | 0.6756 | 0.5078 | 375 | 250 | 0.0167 |
|  | cwgan_gp_0.5x | 0.4186 | 0.4333 | 0.4043 | 0.4173 | 0.6067 | 0.422 | 187 | 62 | 0.0082 |
|  | cwgan_gp_1.0x | 0.5116 | 0.497 | 0.4877 | 0.5236 | 0.6097 | 0.4216 | 250 | 125 | 0.0041 |
|  | cwgan_gp_2.0x | 0.4651 | 0.4182 | 0.422 | 0.4602 | 0.4945 | 0.3698 | 375 | 250 | 0.0082 |
|  | aug_0.5x | 0.5116 | 0.5152 | 0.5082 | 0.5176 | 0.6093 | 0.4713 | 187 | 62 | 0.012 |
|  | aug_1.0x | 0.3721 | 0.4697 | 0.361 | 0.3048 | 0.6024 | 0.4488 | 250 | 125 | 0.0049 |
|  | aug_2.0x | 0.4651 | 0.5182 | 0.469 | 0.4582 | 0.5941 | 0.4533 | 375 | 250 | 0.0052 |
|  | ensemble | 0.4419 | 0.5000 | 0.4401 | 0.4275 | 0.6199 | 0.4429 | NA | NA | NA |
| **48** | baseline | 0.5435 | 0.5524 | 0.5415 | 0.5459 | 0.6864 | 0.5241 | 122 | 0 | 0 |
|  | cvae_0.5x | 0.4565 | 0.446 | 0.4363 | 0.4502 | 0.5823 | 0.4528 | 182 | 60 | 0.0395 |
|  | cvae_1.0x | 0.4348 | 0.4476 | 0.4321 | 0.4253 | 0.5542 | 0.409 | 244 | 122 | 0.0219 |
|  | cvae_2.0x | 0.5217 | 0.5349 | 0.5105 | 0.513 | 0.6077 | 0.4231 | 366 | 244 | 0.0159 |
|  | csdvae_0.5x | 0.4348 | 0.4492 | 0.4345 | 0.4358 | 0.6117 | 0.4598 | 182 | 60 | 0.0402 |
|  | csdvae_1.0x | 0.4783 | 0.4746 | 0.4677 | 0.4786 | 0.5957 | 0.4626 | 244 | 122 | 0.0219 |
|  | csdvae_2.0x | 0.4783 | 0.5095 | 0.4802 | 0.4731 | 0.656 | 0.5036 | 366 | 244 | 0.0172 |
|  | cwgan_gp_0.5x | 0.4565 | 0.5 | 0.4601 | 0.4491 | 0.6068 | 0.4501 | 182 | 60 | 0.0062 |
|  | cwgan_gp_1.0x | 0.4565 | 0.4524 | 0.4487 | 0.4572 | 0.6392 | 0.5092 | 244 | 122 | 0.0044 |
|  | cwgan_gp_2.0x | 0.4565 | 0.5 | 0.4626 | 0.4477 | 0.6638 | 0.5247 | 366 | 244 | 0.0052 |
|  | aug_0.5x | 0.5 | 0.5429 | 0.5026 | 0.4907 | 0.6727 | 0.54 | 182 | 60 | 0.0079 |
|  | aug_1.0x | 0.3913 | 0.4524 | 0.3941 | 0.3648 | 0.6028 | 0.4159 | 244 | 122 | 0.0081 |
|  | aug_2.0x | 0.5 | 0.5619 | 0.5021 | 0.4808 | 0.6294 | 0.4706 | 366 | 244 | 0.0042 |
|  | ensemble | 0.4130 | 0.4317 | 0.4121 | 0.4047 | 0.6109 | 0.4697 | NA | NA | NA |
| **49** | baseline | 0.6154 | 0.5722 | 0.5643 | 0.6237 | 0.7688 | 0.6379 | 116 | 0 | 0 |
|  | cvae_0.5x | 0.5577 | 0.6222 | 0.5453 | 0.5698 | 0.7444 | 0.63 | 173 | 57 | 0.0423 |
|  | cvae_1.0x | 0.4615 | 0.5167 | 0.4365 | 0.4658 | 0.7107 | 0.5897 | 232 | 116 | 0.0323 |
|  | cvae_2.0x | 0.4423 | 0.5333 | 0.4434 | 0.4391 | 0.7283 | 0.6021 | 348 | 232 | 0.0247 |
|  | csdvae_0.5x | 0.5577 | 0.5944 | 0.5404 | 0.5758 | 0.7495 | 0.6358 | 173 | 57 | 0.0481 |
|  | csdvae_1.0x | 0.4231 | 0.5111 | 0.4301 | 0.4178 | 0.7261 | 0.5885 | 232 | 116 | 0.0371 |
|  | csdvae_2.0x | 0.4423 | 0.4722 | 0.4279 | 0.4603 | 0.6975 | 0.5675 | 348 | 232 | 0.0274 |
|  | cwgan_gp_0.5x | 0.4808 | 0.5778 | 0.477 | 0.4846 | 0.7267 | 0.5694 | 173 | 57 | 0.0121 |
|  | cwgan_gp_1.0x | 0.5385 | 0.6 | 0.5303 | 0.5524 | 0.7529 | 0.6101 | 232 | 116 | 0.0128 |
|  | cwgan_gp_2.0x | 0.5577 | 0.6611 | 0.5656 | 0.5573 | 0.8446 | 0.7416 | 348 | 232 | 0.0157 |
|  | aug_0.5x | 0.5 | 0.5722 | 0.4936 | 0.5118 | 0.7123 | 0.5676 | 173 | 57 | 0.0096 |
|  | aug_1.0x | 0.4808 | 0.5889 | 0.5056 | 0.4801 | 0.7075 | 0.5582 | 232 | 116 | 0.0062 |
|  | aug_2.0x | 0.5192 | 0.5944 | 0.5131 | 0.5331 | 0.7701 | 0.5881 | 348 | 232 | 0.0053 |
|  | ensemble | 0.5385 | 0.6500 | 0.5402 | 0.5305 | 0.7601 | 0.6218 | NA | NA | NA |
| **50** | baseline | 0.3729 | 0.5167 | 0.3693 | 0.3314 | 0.5481 | 0.4016 | 109 | 0 | 0 |
|  | cvae_0.5x | 0.339 | 0.4656 | 0.3358 | 0.2851 | 0.6312 | 0.4711 | 163 | 54 | 0.028 |
|  | cvae_1.0x | 0.3051 | 0.4815 | 0.2764 | 0.1976 | 0.5269 | 0.3691 | 218 | 109 | 0.0224 |
|  | cvae_2.0x | 0.3559 | 0.4763 | 0.3505 | 0.3168 | 0.5965 | 0.4208 | 327 | 218 | 0.0212 |
|  | csdvae_0.5x | 0.3559 | 0.4834 | 0.3545 | 0.3228 | 0.5941 | 0.4262 | 163 | 54 | 0.0235 |
|  | csdvae_1.0x | 0.339 | 0.4578 | 0.3355 | 0.3018 | 0.5615 | 0.3919 | 218 | 109 | 0.019 |
|  | csdvae_2.0x | 0.3729 | 0.4949 | 0.3676 | 0.3284 | 0.6166 | 0.4576 | 327 | 218 | 0.018 |
|  | cwgan_gp_0.5x | 0.322 | 0.4852 | 0.3027 | 0.2093 | 0.6352 | 0.4704 | 163 | 54 | 0.013 |
|  | cwgan_gp_1.0x | 0.4237 | 0.5645 | 0.4231 | 0.3889 | 0.6637 | 0.4764 | 218 | 109 | 0.0044 |
|  | cwgan_gp_2.0x | 0.3729 | 0.5478 | 0.3506 | 0.2647 | 0.6094 | 0.4308 | 327 | 218 | 0.0041 |
|  | aug_0.5x | 0.3559 | 0.4834 | 0.3561 | 0.32 | 0.6012 | 0.4313 | 163 | 54 | 0.0102 |
|  | aug_1.0x | 0.3729 | 0.5323 | 0.3619 | 0.3023 | 0.6087 | 0.4569 | 218 | 109 | 0.0055 |
|  | aug_2.0x | 0.322 | 0.4774 | 0.2999 | 0.2315 | 0.6553 | 0.5021 | 327 | 218 | 0.0081 |
|  | ensemble | 0.3559 | 0.5137 | 0.3437 | 0.2932 | 0.6287 | 0.4579 | NA | NA | NA |
| **51** | baseline | 0.4717 | 0.5944 | 0.4107 | 0.4214 | 0.7505 | 0.6376 | 115 | 0 | 0 |
|  | cvae_0.5x | 0.566 | 0.6678 | 0.526 | 0.5468 | 0.7717 | 0.6137 | 172 | 57 | 0.0178 |
|  | cvae_1.0x | 0.5094 | 0.6247 | 0.4691 | 0.4715 | 0.736 | 0.54 | 230 | 115 | 0.0214 |
|  | cvae_2.0x | 0.5094 | 0.5685 | 0.4366 | 0.459 | 0.7165 | 0.5369 | 345 | 230 | 0.0101 |
|  | csdvae_0.5x | 0.4906 | 0.6119 | 0.4537 | 0.4589 | 0.71 | 0.491 | 172 | 57 | 0.0116 |
|  | csdvae_1.0x | 0.5472 | 0.6058 | 0.504 | 0.5731 | 0.7179 | 0.5123 | 230 | 115 | 0.0181 |
|  | csdvae_2.0x | 0.5094 | 0.5709 | 0.4484 | 0.4813 | 0.6553 | 0.4942 | 345 | 230 | 0.006 |
|  | cwgan_gp_0.5x | 0.5094 | 0.6247 | 0.455 | 0.4895 | 0.7548 | 0.5668 | 172 | 57 | 0.0086 |
|  | cwgan_gp_1.0x | 0.5283 | 0.6375 | 0.4817 | 0.4907 | 0.7302 | 0.548 | 230 | 115 | 0.0079 |
|  | cwgan_gp_2.0x | 0.4906 | 0.6072 | 0.4422 | 0.4195 | 0.6895 | 0.5032 | 345 | 230 | 0.0093 |
|  | aug_0.5x | 0.4906 | 0.6119 | 0.4424 | 0.4778 | 0.748 | 0.5742 | 172 | 57 | 0.0041 |
|  | aug_1.0x | 0.434 | 0.5149 | 0.4097 | 0.3819 | 0.6739 | 0.4842 | 230 | 115 | 0.0055 |
|  | aug_2.0x | 0.4528 | 0.5394 | 0.4499 | 0.4485 | 0.6156 | 0.4093 | 345 | 230 | 0.0036 |
|  | ensemble | 0.5283 | 0.6375 | 0.4762 | 0.4906 | 0.7648 | 0.5980 | NA | NA | NA |
| **52** | baseline | 0.4423 | 0.4338 | 0.4109 | 0.4278 | 0.6312 | 0.4815 | 116 | 0 | 0 |
|  | cvae_0.5x | 0.5577 | 0.5518 | 0.5423 | 0.561 | 0.7502 | 0.6118 | 174 | 58 | 0.019 |
|  | cvae_1.0x | 0.5192 | 0.4537 | 0.4353 | 0.4832 | 0.6037 | 0.4886 | 232 | 116 | 0.022 |
|  | cvae_2.0x | 0.4231 | 0.4187 | 0.4034 | 0.4201 | 0.6087 | 0.4637 | 348 | 232 | 0.0167 |
|  | csdvae_0.5x | 0.5385 | 0.4936 | 0.4933 | 0.5372 | 0.6671 | 0.5397 | 174 | 58 | 0.0235 |
|  | csdvae_1.0x | 0.4231 | 0.4155 | 0.4126 | 0.4254 | 0.6094 | 0.488 | 232 | 116 | 0.0274 |
|  | csdvae_2.0x | 0.5 | 0.5215 | 0.4989 | 0.5156 | 0.6398 | 0.505 | 348 | 232 | 0.0213 |
|  | cwgan_gp_0.5x | 0.6154 | 0.6077 | 0.5907 | 0.5984 | 0.6983 | 0.5724 | 174 | 58 | 0.0104 |
|  | cwgan_gp_1.0x | 0.4808 | 0.441 | 0.4375 | 0.4772 | 0.5983 | 0.4482 | 232 | 116 | 0.0067 |
|  | cwgan_gp_2.0x | 0.4615 | 0.4537 | 0.441 | 0.4626 | 0.6233 | 0.4821 | 348 | 232 | 0.0075 |
|  | aug_0.5x | 0.4423 | 0.4163 | 0.3872 | 0.4114 | 0.6481 | 0.4972 | 174 | 58 | 0.0099 |
|  | aug_1.0x | 0.6154 | 0.5797 | 0.5694 | 0.6048 | 0.749 | 0.6093 | 232 | 116 | 0.0056 |
|  | aug_2.0x | 0.5385 | 0.504 | 0.5016 | 0.5417 | 0.7027 | 0.5698 | 348 | 232 | 0.0109 |
|  | ensemble | 0.5385 | 0.4713 | 0.4562 | 0.5107 | 0.6854 | 0.5358 | NA | NA | NA |
