# Supplementary material for: Synthetic data augmentation for CT-based emphysema subtype classification: A comparative evaluation of generative and classical approaches
Source: PLoS One. 2026 Aug 20;21(8):e0355850. doi: 10.1371/journal.pone.0355850 (PMC13492795; doi:10.1371/journal.pone.0355850)
Supplement: Supplementary file 4 [file pone.0355850.s004.docx]

**S3 Table.**

| **Method** | **Metric** | **Mean Baseline** | **Mean Method** | **Difference** | **Wilcoxon_P (uncorrected)** | **Wilcoxon_P (corrected)** | **Cohens_d** |
| --- | --- | --- | --- | --- | --- | --- | --- |
| **cvae_0.5x** | balanced_accuracy | 0.5224 | 0.5161 | -0.0063 | 0.8457 | 1 | -0.087 |
| **cvae_1.0x** | balanced_accuracy | 0.5224 | 0.5133 | -0.0091 | 0.7695 | 1 | -0.173 |
| **cvae_2.0x** | balanced_accuracy | 0.5224 | 0.5087 | -0.0137 | 0.3223 | 1 | -0.336 |
| **csdvae_0.5x** | balanced_accuracy | 0.5224 | 0.5239 | 0.0015 | 0.7695 | 1 | 0.034 |
| **csdvae_1.0x** | balanced_accuracy | 0.5224 | 0.503 | -0.0194 | 0.3223 | 1 | -0.48 |
| **csdvae_2.0x** | balanced_accuracy | 0.5224 | 0.508 | -0.0143 | 0.4922 | 1 | -0.233 |
| **cwgan_gp_0.5x** | balanced_accuracy | 0.5224 | 0.525 | 0.0027 | 0.6953 | 1 | 0.04 |
| **cwgan_gp_1.0x** | balanced_accuracy | 0.5224 | 0.5483 | 0.0259 | 0.084 | 1 | 0.47 |
| **cwgan_gp_2.0x** | balanced_accuracy | 0.5224 | 0.4944 | -0.028 | 0.2324 | 1 | -0.393 |
| **aug_0.5x** | balanced_accuracy | 0.5224 | 0.5277 | 0.0053 | 1 | 1 | 0.167 |
| **aug_1.0x** | balanced_accuracy | 0.5224 | 0.5196 | -0.0028 | 0.6953 | 1 | -0.043 |
| **aug_2.0x** | balanced_accuracy | 0.5224 | 0.523 | 0.0006 | 1 | 1 | 0.013 |
| **ensemble** | balanced_accuracy | 0.5224 | 0.5925 | 0.0701 | 0.002 | 1 | 1.23 |
| **cvae_0.5x** | auroc_macro | 0.6758 | 0.6635 | -0.0123 | 0.4922 | 1 | -0.179 |
| **cvae_1.0x** | auroc_macro | 0.6758 | 0.6387 | -0.0371 | 0.0273 | 0.3555 | -0.906 |
| **cvae_2.0x** | auroc_macro | 0.6758 | 0.6432 | -0.0326 | 0.1309 | 1 | -0.61 |
| **csdvae_0.5x** | auroc_macro | 0.6758 | 0.6638 | -0.0121 | 0.4922 | 1 | -0.307 |
| **csdvae_1.0x** | auroc_macro | 0.6758 | 0.6407 | -0.0351 | 0.0195 | 0.2539 | -0.983 |
| **csdvae_2.0x** | auroc_macro | 0.6758 | 0.6551 | -0.0207 | 0.2754 | 1 | -0.368 |
| **cwgan_gp_0.5x** | auroc_macro | 0.6758 | 0.6669 | -0.0089 | 0.7695 | 1 | -0.166 |
| **cwgan_gp_1.0x** | auroc_macro | 0.6758 | 0.6753 | -0.0005 | 0.4316 | 1 | -0.013 |
| **cwgan_gp_2.0x** | auroc_macro | 0.6758 | 0.6427 | -0.0331 | 0.1602 | 1 | -0.523 |
| **aug_0.5x** | auroc_macro | 0.6758 | 0.6711 | -0.0047 | 0.7695 | 1 | -0.129 |
| **aug_1.0x** | auroc_macro | 0.6758 | 0.6577 | -0.0181 | 0.3223 | 1 | -0.278 |
| **aug_2.0x** | auroc_macro | 0.6758 | 0.6597 | -0.0161 | 0.5566 | 1 | -0.222 |
| **ensemble** | auroc_macro | 0.6758 | 0.7377 | 0.0619 | 0.002 | 1 | 1.392 |
| **cvae_0.5x** | f1_macro | 0.4428 | 0.4389 | -0.0039 | 0.8457 | 1 | -0.051 |
| **cvae_1.0x** | f1_macro | 0.4428 | 0.4189 | -0.024 | 0.4316 | 1 | -0.386 |
| **cvae_2.0x** | f1_macro | 0.4428 | 0.4184 | -0.0245 | 0.1934 | 1 | -0.485 |
| **csdvae_0.5x** | f1_macro | 0.4428 | 0.4367 | -0.0061 | 0.8457 | 1 | -0.107 |
| **csdvae_1.0x** | f1_macro | 0.4428 | 0.4347 | -0.0081 | 0.9219 | 1 | -0.112 |
| **csdvae_2.0x** | f1_macro | 0.4428 | 0.4362 | -0.0066 | 0.8457 | 1 | -0.082 |
| **cwgan_gp_0.5x** | f1_macro | 0.4428 | 0.4417 | -0.0011 | 0.7695 | 1 | -0.014 |
| **cwgan_gp_1.0x** | f1_macro | 0.4428 | 0.472 | 0.0292 | 0.2324 | 1 | 0.431 |
| **cwgan_gp_2.0x** | f1_macro | 0.4428 | 0.4088 | -0.034 | 0.3223 | 1 | -0.518 |
| **aug_0.5x** | f1_macro | 0.4428 | 0.4299 | -0.0129 | 0.375 | 1 | -0.233 |
| **aug_1.0x** | f1_macro | 0.4428 | 0.4176 | -0.0252 | 0.084 | 1 | -0.337 |
| **aug_2.0x** | f1_macro | 0.4428 | 0.445 | 0.0021 | 0.9219 | 1 | 0.038 |
| **ensemble** | f1_macro | 0.4428 | 0.4935 | 0.0507 | 0.0371 | 1 | 0.895 |
| **cvae_0.5x** | auprc_macro | 0.5362 | 0.5227 | -0.0135 | 0.4316 | 1 | -0.204 |
| **cvae_1.0x** | auprc_macro | 0.5362 | 0.4849 | -0.0513 | 0.0098 | 0.127 | -1.2 |
| **cvae_2.0x** | auprc_macro | 0.5362 | 0.4735 | -0.0626 | 0.0273 | 0.3555 | -0.866 |
| **csdvae_0.5x** | auprc_macro | 0.5362 | 0.5065 | -0.0296 | 0.2754 | 1 | -0.473 |
| **csdvae_1.0x** | auprc_macro | 0.5362 | 0.4876 | -0.0486 | 0.0137 | 0.1777 | -1.064 |
| **csdvae_2.0x** | auprc_macro | 0.5362 | 0.5041 | -0.0321 | 0.2754 | 1 | -0.484 |
| **cwgan_gp_0.5x** | auprc_macro | 0.5362 | 0.5102 | -0.026 | 0.3223 | 1 | -0.376 |
| **cwgan_gp_1.0x** | auprc_macro | 0.5362 | 0.519 | -0.0172 | 0.2324 | 1 | -0.401 |
| **cwgan_gp_2.0x** | auprc_macro | 0.5362 | 0.4928 | -0.0434 | 0.1934 | 1 | -0.578 |
| **aug_0.5x** | auprc_macro | 0.5362 | 0.5241 | -0.0121 | 0.6953 | 1 | -0.243 |
| **aug_1.0x** | auprc_macro | 0.5362 | 0.4982 | -0.038 | 0.2324 | 1 | -0.453 |
| **aug_2.0x** | auprc_macro | 0.5362 | 0.4911 | -0.0451 | 0.1934 | 1 | -0.475 |
| **ensemble** | auprc_macro | 0.5362 | 0.5885 | 0.0523 | 0.0273 | 1 | 0.852 |
