# Supplementary material for: Synthetic data augmentation for CT-based emphysema subtype classification: A comparative evaluation of generative and classical approaches
Source: PLoS One. 2026 Aug 20;21(8):e0355850. doi: 10.1371/journal.pone.0355850 (PMC13492795; doi:10.1371/journal.pone.0355850)
Supplement: Supplementary file 5 [file pone.0355850.s005.docx]

**S4 Table.**

| **Method** | **Mean** | **SD** | **Diff** | **Cohens d** | **P uncorrected** | **P Bonferroni** |
| --- | --- | --- | --- | --- | --- | --- |
| **cvae_0.5x** | 0.2904 | 0.166 | 0.0338 | 0.268 | 0.4961 | 1 |
| **cvae_1.0x** | 0.2898 | 0.2209 | 0.0332 | 0.199 | 0.9102 | 1 |
| **cvae_2.0x** | 0.2687 | 0.1972 | 0.0121 | 0.169 | 0.5469 | 1 |
| **csdvae_0.5x** | 0.2912 | 0.1831 | 0.0346 | 0.245 | 0.6406 | 1 |
| **csdvae_1.0x** | 0.3615 | 0.1642 | 0.1049 | 0.749 | 0.0781 | 1 |
| **csdvae_2.0x** | 0.3211 | 0.1525 | 0.0644 | 0.51 | 0.2031 | 1 |
| **cwgan_gp_0.5x** | 0.2927 | 0.1453 | 0.036 | 0.257 | 0.6523 | 1 |
| **cwgan_gp_1.0x** | 0.3846 | 0.2086 | 0.128 | 0.723 | 0.0742 | 0.9648 |
| **cwgan_gp_2.0x** | 0.2601 | 0.1967 | 0.0035 | 0.02 | 0.9102 | 1 |
| **aug_0.5x** | 0.2814 | 0.2103 | 0.0248 | 0.161 | 0.8438 | 1 |
| **aug_1.0x** | 0.2027 | 0.1931 | -0.0539 | -0.313 | 0.3008 | 1 |
| **aug_2.0x** | 0.281 | 0.1466 | 0.0244 | 0.149 | 0.7695 | 1 |
| **ensemble** | 0.3208 | 0.1948 | 0.0642 | 0.489 | 0.1934 | 1 |
