# Supplementary material for: Synthetic data augmentation for CT-based emphysema subtype classification: A comparative evaluation of generative and classical approaches
Source: PLoS One. 2026 Aug 20;21(8):e0355850. doi: 10.1371/journal.pone.0355850 (PMC13492795; doi:10.1371/journal.pone.0355850)
Supplement: Supplementary file 6 [file pone.0355850.s006.docx]

**S5 Table.**

| **Seed** | **Method** | **Class** | **Sensitivity** | **Specificity** | **Precision** | **NPV** | **F1** | **Support** |
| --- | --- | --- | --- | --- | --- | --- | --- | --- |
| **43** | baseline | Normal | 0.9375 | 0.4773 | 0.3947 | 0.9545 | 0.5556 | 16 |
|  | baseline | CLE | 0.1034 | 0.9355 | 0.6 | 0.5273 | 0.1765 | 29 |
|  | baseline | PSE | 0.7333 | 0.8667 | 0.6471 | 0.907 | 0.6875 | 15 |
|  | cvae_0.5x | Normal | 0.6875 | 0.5227 | 0.3438 | 0.8214 | 0.4583 | 16 |
|  | cvae_0.5x | CLE | 0.1379 | 0.8065 | 0.4 | 0.5 | 0.2051 | 29 |
|  | cvae_0.5x | PSE | 0.6 | 0.8 | 0.5 | 0.8571 | 0.5455 | 15 |
|  | cvae_1.0x | Normal | 0.625 | 0.6136 | 0.3704 | 0.8182 | 0.4651 | 16 |
|  | cvae_1.0x | CLE | 0.2414 | 0.8065 | 0.5385 | 0.5319 | 0.3333 | 29 |
|  | cvae_1.0x | PSE | 0.6667 | 0.7778 | 0.5 | 0.875 | 0.5714 | 15 |
|  | cvae_2.0x | Normal | 0.75 | 0.75 | 0.5217 | 0.8919 | 0.6154 | 16 |
|  | cvae_2.0x | CLE | 0 | 0.9032 | 0 | 0.4912 | 0 | 29 |
|  | cvae_2.0x | PSE | 0.8 | 0.5111 | 0.3529 | 0.8846 | 0.4898 | 15 |
|  | csdvae_0.5x | Normal | 0.8125 | 0.6364 | 0.4483 | 0.9032 | 0.5778 | 16 |
|  | csdvae_0.5x | CLE | 0 | 0.9677 | 0 | 0.5085 | 0 | 29 |
|  | csdvae_0.5x | PSE | 0.9333 | 0.6444 | 0.4667 | 0.9667 | 0.6222 | 15 |
|  | csdvae_1.0x | Normal | 0.75 | 0.6364 | 0.4286 | 0.875 | 0.5455 | 16 |
|  | csdvae_1.0x | CLE | 0.3103 | 0.8065 | 0.6 | 0.5556 | 0.4091 | 29 |
|  | csdvae_1.0x | PSE | 0.7333 | 0.8667 | 0.6471 | 0.907 | 0.6875 | 15 |
|  | csdvae_2.0x | Normal | 0.8125 | 0.7727 | 0.5652 | 0.9189 | 0.6667 | 16 |
|  | csdvae_2.0x | CLE | 0.2759 | 0.8387 | 0.6154 | 0.5532 | 0.381 | 29 |
|  | csdvae_2.0x | PSE | 0.8 | 0.7333 | 0.5 | 0.9167 | 0.6154 | 15 |
|  | cwgan_gp_0.5x | Normal | 0.6875 | 0.5909 | 0.3793 | 0.8387 | 0.4889 | 16 |
|  | cwgan_gp_0.5x | CLE | 0.2414 | 0.8387 | 0.5833 | 0.5417 | 0.3415 | 29 |
|  | cwgan_gp_0.5x | PSE | 0.8 | 0.8444 | 0.6316 | 0.9268 | 0.7059 | 15 |
|  | cwgan_gp_1.0x | Normal | 0.5625 | 0.75 | 0.45 | 0.825 | 0.5 | 16 |
|  | cwgan_gp_1.0x | CLE | 0.5862 | 0.7419 | 0.68 | 0.6571 | 0.6296 | 29 |
|  | cwgan_gp_1.0x | PSE | 0.7333 | 0.9111 | 0.7333 | 0.9111 | 0.7333 | 15 |
|  | cwgan_gp_2.0x | Normal | 0.75 | 0.5682 | 0.3871 | 0.8621 | 0.5106 | 16 |
|  | cwgan_gp_2.0x | CLE | 0.1379 | 0.9032 | 0.5714 | 0.5283 | 0.2222 | 29 |
|  | cwgan_gp_2.0x | PSE | 0.7333 | 0.7556 | 0.5 | 0.8947 | 0.5946 | 15 |
|  | aug_0.5x | Normal | 0.6875 | 0.6818 | 0.44 | 0.8571 | 0.5366 | 16 |
|  | aug_0.5x | CLE | 0.1379 | 0.7742 | 0.3636 | 0.4898 | 0.2 | 29 |
|  | aug_0.5x | PSE | 0.8667 | 0.7556 | 0.5417 | 0.9444 | 0.6667 | 15 |
|  | aug_1.0x | Normal | 0.875 | 0.6136 | 0.4516 | 0.931 | 0.5957 | 16 |
|  | aug_1.0x | CLE | 0.069 | 0.9677 | 0.6667 | 0.5263 | 0.125 | 29 |
|  | aug_1.0x | PSE | 0.9333 | 0.7333 | 0.5385 | 0.9706 | 0.6829 | 15 |
|  | aug_2.0x | Normal | 0.75 | 0.7727 | 0.5455 | 0.8947 | 0.6316 | 16 |
|  | aug_2.0x | CLE | 0.2414 | 0.7742 | 0.5 | 0.5217 | 0.3256 | 29 |
|  | aug_2.0x | PSE | 0.7333 | 0.7111 | 0.4583 | 0.8889 | 0.5641 | 15 |
|  | ensemble | Normal | 0.625 | 0.5909 | 0.3571 | 0.8125 | 0.4545 | 16 |
|  | ensemble | CLE | 0.2759 | 0.8065 | 0.5714 | 0.5435 | 0.3721 | 29 |
|  | ensemble | PSE | 0.8 | 0.8667 | 0.6667 | 0.9286 | 0.7273 | 15 |
| **44** | baseline | Normal | 0.6071 | 0.9333 | 0.8947 | 0.7179 | 0.7234 | 28 |
|  | baseline | CLE | 0.4286 | 0.7843 | 0.2143 | 0.9091 | 0.2857 | 7 |
|  | baseline | PSE | 0.7391 | 0.7714 | 0.68 | 0.8182 | 0.7083 | 23 |
|  | cvae_0.5x | Normal | 0.5 | 0.9 | 0.8235 | 0.6585 | 0.6222 | 28 |
|  | cvae_0.5x | CLE | 0.2857 | 0.8431 | 0.2 | 0.8958 | 0.2353 | 7 |
|  | cvae_0.5x | PSE | 0.913 | 0.7143 | 0.6774 | 0.9259 | 0.7778 | 23 |
|  | cvae_1.0x | Normal | 0.4643 | 0.9333 | 0.8667 | 0.6512 | 0.6047 | 28 |
|  | cvae_1.0x | CLE | 0.8571 | 0.6078 | 0.2308 | 0.9688 | 0.3636 | 7 |
|  | cvae_1.0x | PSE | 0.5652 | 0.8857 | 0.7647 | 0.7561 | 0.65 | 23 |
|  | cvae_2.0x | Normal | 0.5 | 0.8667 | 0.7778 | 0.65 | 0.6087 | 28 |
|  | cvae_2.0x | CLE | 0.5714 | 0.7647 | 0.25 | 0.9286 | 0.3478 | 7 |
|  | cvae_2.0x | PSE | 0.6957 | 0.7714 | 0.6667 | 0.7941 | 0.6809 | 23 |
|  | csdvae_0.5x | Normal | 0.5714 | 0.8333 | 0.7619 | 0.6757 | 0.6531 | 28 |
|  | csdvae_0.5x | CLE | 0.4286 | 0.8431 | 0.2727 | 0.9149 | 0.3333 | 7 |
|  | csdvae_0.5x | PSE | 0.7826 | 0.7714 | 0.6923 | 0.8438 | 0.7347 | 23 |
|  | csdvae_1.0x | Normal | 0.3214 | 0.8667 | 0.6923 | 0.5778 | 0.439 | 28 |
|  | csdvae_1.0x | CLE | 0.4286 | 0.6863 | 0.1579 | 0.8974 | 0.2308 | 7 |
|  | csdvae_1.0x | PSE | 0.8261 | 0.8 | 0.7308 | 0.875 | 0.7755 | 23 |
|  | csdvae_2.0x | Normal | 0.3929 | 0.7667 | 0.6111 | 0.575 | 0.4783 | 28 |
|  | csdvae_2.0x | CLE | 0.5714 | 0.549 | 0.1481 | 0.9032 | 0.2353 | 7 |
|  | csdvae_2.0x | PSE | 0.4783 | 0.9429 | 0.8462 | 0.7333 | 0.6111 | 23 |
|  | cwgan_gp_0.5x | Normal | 0.3929 | 0.8667 | 0.7333 | 0.6047 | 0.5116 | 28 |
|  | cwgan_gp_0.5x | CLE | 0.4286 | 0.6863 | 0.1579 | 0.8974 | 0.2308 | 7 |
|  | cwgan_gp_0.5x | PSE | 0.6957 | 0.7714 | 0.6667 | 0.7941 | 0.6809 | 23 |
|  | cwgan_gp_1.0x | Normal | 0.5714 | 0.8667 | 0.8 | 0.6842 | 0.6667 | 28 |
|  | cwgan_gp_1.0x | CLE | 0.5714 | 0.7647 | 0.25 | 0.9286 | 0.3478 | 7 |
|  | cwgan_gp_1.0x | PSE | 0.6957 | 0.8286 | 0.7273 | 0.8056 | 0.7111 | 23 |
|  | cwgan_gp_2.0x | Normal | 0.3929 | 0.9333 | 0.8462 | 0.6222 | 0.5366 | 28 |
|  | cwgan_gp_2.0x | CLE | 0.2857 | 0.5098 | 0.0741 | 0.8387 | 0.1176 | 7 |
|  | cwgan_gp_2.0x | PSE | 0.5217 | 0.8286 | 0.6667 | 0.725 | 0.5854 | 23 |
|  | aug_0.5x | Normal | 0.2857 | 0.9 | 0.7273 | 0.5745 | 0.4103 | 28 |
|  | aug_0.5x | CLE | 0.7143 | 0.7059 | 0.25 | 0.9474 | 0.3704 | 7 |
|  | aug_0.5x | PSE | 0.7826 | 0.7429 | 0.6667 | 0.8387 | 0.72 | 23 |
|  | aug_1.0x | Normal | 0.5 | 0.9667 | 0.9333 | 0.6744 | 0.6512 | 28 |
|  | aug_1.0x | CLE | 0.2857 | 0.8039 | 0.1667 | 0.8913 | 0.2105 | 7 |
|  | aug_1.0x | PSE | 0.913 | 0.7143 | 0.6774 | 0.9259 | 0.7778 | 23 |
|  | aug_2.0x | Normal | 0.5 | 0.9333 | 0.875 | 0.6667 | 0.6364 | 28 |
|  | aug_2.0x | CLE | 0.2857 | 0.7843 | 0.1538 | 0.8889 | 0.2 | 7 |
|  | aug_2.0x | PSE | 0.7391 | 0.6571 | 0.5862 | 0.7931 | 0.6538 | 23 |
|  | ensemble | Normal | 0.5 | 0.9667 | 0.9333 | 0.6744 | 0.6512 | 28 |
|  | ensemble | CLE | 0.7143 | 0.7647 | 0.2941 | 0.9512 | 0.4167 | 7 |
|  | ensemble | PSE | 0.8696 | 0.8286 | 0.7692 | 0.9062 | 0.8163 | 23 |
| **45** | baseline | Normal | 0.2857 | 0.8718 | 0.5455 | 0.6939 | 0.375 | 21 |
|  | baseline | CLE | 0.25 | 0.8571 | 0.6667 | 0.5 | 0.3636 | 32 |
|  | baseline | PSE | 1 | 0.434 | 0.1892 | 1 | 0.3182 | 7 |
|  | cvae_0.5x | Normal | 0.2857 | 0.8462 | 0.5 | 0.6875 | 0.3636 | 21 |
|  | cvae_0.5x | CLE | 0.0938 | 0.8929 | 0.5 | 0.463 | 0.1579 | 32 |
|  | cvae_0.5x | PSE | 1 | 0.3396 | 0.1667 | 1 | 0.2857 | 7 |
|  | cvae_1.0x | Normal | 0.4286 | 0.6923 | 0.4286 | 0.6923 | 0.4286 | 21 |
|  | cvae_1.0x | CLE | 0.1562 | 0.9643 | 0.8333 | 0.5 | 0.2632 | 32 |
|  | cvae_1.0x | PSE | 1 | 0.5094 | 0.2121 | 1 | 0.35 | 7 |
|  | cvae_2.0x | Normal | 0.4762 | 0.8462 | 0.625 | 0.75 | 0.5405 | 21 |
|  | cvae_2.0x | CLE | 0.2188 | 0.8571 | 0.6364 | 0.4898 | 0.3256 | 32 |
|  | cvae_2.0x | PSE | 1 | 0.5094 | 0.2121 | 1 | 0.35 | 7 |
|  | csdvae_0.5x | Normal | 0.381 | 0.7692 | 0.4706 | 0.6977 | 0.4211 | 21 |
|  | csdvae_0.5x | CLE | 0.0938 | 0.9286 | 0.6 | 0.4727 | 0.1622 | 32 |
|  | csdvae_0.5x | PSE | 1 | 0.4151 | 0.1842 | 1 | 0.3111 | 7 |
|  | csdvae_1.0x | Normal | 0.4762 | 0.7949 | 0.5556 | 0.7381 | 0.5128 | 21 |
|  | csdvae_1.0x | CLE | 0.0938 | 0.8571 | 0.4286 | 0.4528 | 0.1538 | 32 |
|  | csdvae_1.0x | PSE | 1 | 0.4717 | 0.2 | 1 | 0.3333 | 7 |
|  | csdvae_2.0x | Normal | 0.4286 | 0.8205 | 0.5625 | 0.7273 | 0.4865 | 21 |
|  | csdvae_2.0x | CLE | 0.0938 | 1 | 1 | 0.4912 | 0.1714 | 32 |
|  | csdvae_2.0x | PSE | 1 | 0.3585 | 0.1707 | 1 | 0.2917 | 7 |
|  | cwgan_gp_0.5x | Normal | 0.4762 | 0.7692 | 0.5263 | 0.7317 | 0.5 | 21 |
|  | cwgan_gp_0.5x | CLE | 0.125 | 0.8929 | 0.5714 | 0.4717 | 0.2051 | 32 |
|  | cwgan_gp_0.5x | PSE | 1 | 0.4906 | 0.2059 | 1 | 0.3415 | 7 |
|  | cwgan_gp_1.0x | Normal | 0.4286 | 0.7949 | 0.5294 | 0.7209 | 0.4737 | 21 |
|  | cwgan_gp_1.0x | CLE | 0.0938 | 0.9643 | 0.75 | 0.4821 | 0.1667 | 32 |
|  | cwgan_gp_1.0x | PSE | 1 | 0.3962 | 0.1795 | 1 | 0.3043 | 7 |
|  | cwgan_gp_2.0x | Normal | 0.3333 | 0.7179 | 0.3889 | 0.6667 | 0.359 | 21 |
|  | cwgan_gp_2.0x | CLE | 0 | 1 | 0 | 0.4667 | 0 | 32 |
|  | cwgan_gp_2.0x | PSE | 1 | 0.3396 | 0.1667 | 1 | 0.2857 | 7 |
|  | aug_0.5x | Normal | 0.5238 | 0.7436 | 0.5238 | 0.7436 | 0.5238 | 21 |
|  | aug_0.5x | CLE | 0 | 0.9286 | 0 | 0.4483 | 0 | 32 |
|  | aug_0.5x | PSE | 1 | 0.434 | 0.1892 | 1 | 0.3182 | 7 |
|  | aug_1.0x | Normal | 0.4762 | 0.6667 | 0.4348 | 0.7027 | 0.4545 | 21 |
|  | aug_1.0x | CLE | 0 | 0.9643 | 0 | 0.4576 | 0 | 32 |
|  | aug_1.0x | PSE | 1 | 0.4528 | 0.1944 | 1 | 0.3256 | 7 |
|  | aug_2.0x | Normal | 0.5714 | 0.6667 | 0.48 | 0.7429 | 0.5217 | 21 |
|  | aug_2.0x | CLE | 0.0938 | 0.9286 | 0.6 | 0.4727 | 0.1622 | 32 |
|  | aug_2.0x | PSE | 1 | 0.566 | 0.2333 | 1 | 0.3784 | 7 |
|  | ensemble | Normal | 0.4286 | 0.7436 | 0.4737 | 0.7073 | 0.45 | 21 |
|  | ensemble | CLE | 0.0938 | 0.9286 | 0.6 | 0.4727 | 0.1622 | 32 |
|  | ensemble | PSE | 1 | 0.4528 | 0.1944 | 1 | 0.3256 | 7 |
| **46** | baseline | Normal | 0.625 | 0.5429 | 0.3846 | 0.76 | 0.4762 | 16 |
|  | baseline | CLE | 0.1538 | 0.8 | 0.4444 | 0.4762 | 0.2286 | 26 |
|  | baseline | PSE | 0.4444 | 0.7143 | 0.25 | 0.8571 | 0.32 | 9 |
|  | cvae_0.5x | Normal | 0.5625 | 0.6 | 0.3913 | 0.75 | 0.4615 | 16 |
|  | cvae_0.5x | CLE | 0.1538 | 0.8 | 0.4444 | 0.4762 | 0.2286 | 26 |
|  | cvae_0.5x | PSE | 0.5556 | 0.6667 | 0.2632 | 0.875 | 0.3571 | 9 |
|  | cvae_1.0x | Normal | 0.5 | 0.6571 | 0.4 | 0.7419 | 0.4444 | 16 |
|  | cvae_1.0x | CLE | 0.1923 | 0.8 | 0.5 | 0.4878 | 0.2778 | 26 |
|  | cvae_1.0x | PSE | 0.6667 | 0.6429 | 0.2857 | 0.9 | 0.4 | 9 |
|  | cvae_2.0x | Normal | 0.5625 | 0.6 | 0.3913 | 0.75 | 0.4615 | 16 |
|  | cvae_2.0x | CLE | 0.0769 | 0.88 | 0.4 | 0.4783 | 0.129 | 26 |
|  | cvae_2.0x | PSE | 0.7778 | 0.619 | 0.3043 | 0.9286 | 0.4375 | 9 |
|  | csdvae_0.5x | Normal | 0.4375 | 0.5714 | 0.3182 | 0.6897 | 0.3684 | 16 |
|  | csdvae_0.5x | CLE | 0.1923 | 0.88 | 0.625 | 0.5116 | 0.2941 | 26 |
|  | csdvae_0.5x | PSE | 0.7778 | 0.6667 | 0.3333 | 0.9333 | 0.4667 | 9 |
|  | csdvae_1.0x | Normal | 0.4375 | 0.6571 | 0.3684 | 0.7188 | 0.4 | 16 |
|  | csdvae_1.0x | CLE | 0.3077 | 0.68 | 0.5 | 0.4857 | 0.381 | 26 |
|  | csdvae_1.0x | PSE | 0.5556 | 0.7381 | 0.3125 | 0.8857 | 0.4 | 9 |
|  | csdvae_2.0x | Normal | 0.5625 | 0.6286 | 0.4091 | 0.7586 | 0.4737 | 16 |
|  | csdvae_2.0x | CLE | 0.2308 | 0.68 | 0.4286 | 0.4595 | 0.3 | 26 |
|  | csdvae_2.0x | PSE | 0.3333 | 0.7143 | 0.2 | 0.8333 | 0.25 | 9 |
|  | cwgan_gp_0.5x | Normal | 0.5 | 0.6 | 0.3636 | 0.7241 | 0.4211 | 16 |
|  | cwgan_gp_0.5x | CLE | 0.3846 | 0.68 | 0.5556 | 0.5152 | 0.4545 | 26 |
|  | cwgan_gp_0.5x | PSE | 0.3333 | 0.8095 | 0.2727 | 0.85 | 0.3 | 9 |
|  | cwgan_gp_1.0x | Normal | 0.625 | 0.5429 | 0.3846 | 0.76 | 0.4762 | 16 |
|  | cwgan_gp_1.0x | CLE | 0.1154 | 1 | 1 | 0.5208 | 0.2069 | 26 |
|  | cwgan_gp_1.0x | PSE | 0.8889 | 0.6667 | 0.3636 | 0.9655 | 0.5161 | 9 |
|  | cwgan_gp_2.0x | Normal | 0.4375 | 0.6286 | 0.35 | 0.7097 | 0.3889 | 16 |
|  | cwgan_gp_2.0x | CLE | 0.2308 | 0.64 | 0.4 | 0.4444 | 0.2927 | 26 |
|  | cwgan_gp_2.0x | PSE | 0.4444 | 0.7143 | 0.25 | 0.8571 | 0.32 | 9 |
|  | aug_0.5x | Normal | 0.625 | 0.6 | 0.4167 | 0.7778 | 0.5 | 16 |
|  | aug_0.5x | CLE | 0.1154 | 0.92 | 0.6 | 0.5 | 0.1935 | 26 |
|  | aug_0.5x | PSE | 0.6667 | 0.619 | 0.2727 | 0.8966 | 0.3871 | 9 |
|  | aug_1.0x | Normal | 0.5625 | 0.4286 | 0.3103 | 0.6818 | 0.4 | 16 |
|  | aug_1.0x | CLE | 0.1154 | 0.92 | 0.6 | 0.5 | 0.1935 | 26 |
|  | aug_1.0x | PSE | 0.4444 | 0.6905 | 0.2353 | 0.8529 | 0.3077 | 9 |
|  | aug_2.0x | Normal | 0.625 | 0.6286 | 0.4348 | 0.7857 | 0.5128 | 16 |
|  | aug_2.0x | CLE | 0.2308 | 0.76 | 0.5 | 0.4872 | 0.3158 | 26 |
|  | aug_2.0x | PSE | 0.3333 | 0.6905 | 0.1875 | 0.8286 | 0.24 | 9 |
|  | ensemble | Normal | 0.5625 | 0.6 | 0.3913 | 0.75 | 0.4615 | 16 |
|  | ensemble | CLE | 0.1923 | 0.84 | 0.5556 | 0.5 | 0.2857 | 26 |
|  | ensemble | PSE | 0.5556 | 0.6667 | 0.2632 | 0.875 | 0.3571 | 9 |
| **47** | baseline | Normal | 0.4 | 0.7576 | 0.3333 | 0.8065 | 0.3636 | 10 |
|  | baseline | CLE | 0.2273 | 0.7619 | 0.5 | 0.4848 | 0.3125 | 22 |
|  | baseline | PSE | 0.7273 | 0.5938 | 0.381 | 0.8636 | 0.5 | 11 |
|  | cvae_0.5x | Normal | 0.4 | 0.8182 | 0.4 | 0.8182 | 0.4 | 10 |
|  | cvae_0.5x | CLE | 0.3182 | 0.7619 | 0.5833 | 0.5161 | 0.4118 | 22 |
|  | cvae_0.5x | PSE | 0.7273 | 0.5938 | 0.381 | 0.8636 | 0.5 | 11 |
|  | cvae_1.0x | Normal | 0.6 | 0.6061 | 0.3158 | 0.8333 | 0.4138 | 10 |
|  | cvae_1.0x | CLE | 0.2273 | 0.8571 | 0.625 | 0.5143 | 0.3333 | 22 |
|  | cvae_1.0x | PSE | 0.6364 | 0.7188 | 0.4375 | 0.8519 | 0.5185 | 11 |
|  | cvae_2.0x | Normal | 0.6 | 0.7273 | 0.4 | 0.8571 | 0.48 | 10 |
|  | cvae_2.0x | CLE | 0.2727 | 0.6667 | 0.4615 | 0.4667 | 0.3429 | 22 |
|  | cvae_2.0x | PSE | 0.3636 | 0.6562 | 0.2667 | 0.75 | 0.3077 | 11 |
|  | csdvae_0.5x | Normal | 0.5 | 0.7879 | 0.4167 | 0.8387 | 0.4545 | 10 |
|  | csdvae_0.5x | CLE | 0.3636 | 0.7143 | 0.5714 | 0.5172 | 0.4444 | 22 |
|  | csdvae_0.5x | PSE | 0.5455 | 0.6562 | 0.3529 | 0.8077 | 0.4286 | 11 |
|  | csdvae_1.0x | Normal | 0.5 | 0.697 | 0.3333 | 0.8214 | 0.4 | 10 |
|  | csdvae_1.0x | CLE | 0.3182 | 0.8095 | 0.6364 | 0.5312 | 0.4242 | 22 |
|  | csdvae_1.0x | PSE | 0.6364 | 0.6875 | 0.4118 | 0.8462 | 0.5 | 11 |
|  | csdvae_2.0x | Normal | 0.6 | 0.7273 | 0.4 | 0.8571 | 0.48 | 10 |
|  | csdvae_2.0x | CLE | 0.4091 | 0.8095 | 0.6923 | 0.5667 | 0.5143 | 22 |
|  | csdvae_2.0x | PSE | 0.5455 | 0.7188 | 0.4 | 0.8214 | 0.4615 | 11 |
|  | cwgan_gp_0.5x | Normal | 0.3 | 0.7576 | 0.2727 | 0.7812 | 0.2857 | 10 |
|  | cwgan_gp_0.5x | CLE | 0.3636 | 0.7143 | 0.5714 | 0.5172 | 0.4444 | 22 |
|  | cwgan_gp_0.5x | PSE | 0.6364 | 0.6562 | 0.3889 | 0.84 | 0.4828 | 11 |
|  | cwgan_gp_1.0x | Normal | 0.4 | 0.7273 | 0.3077 | 0.8 | 0.3478 | 10 |
|  | cwgan_gp_1.0x | CLE | 0.5455 | 0.7619 | 0.7059 | 0.6154 | 0.6154 | 22 |
|  | cwgan_gp_1.0x | PSE | 0.5455 | 0.7812 | 0.4615 | 0.8333 | 0.5 | 11 |
|  | cwgan_gp_2.0x | Normal | 0.3 | 0.8788 | 0.4286 | 0.8056 | 0.3529 | 10 |
|  | cwgan_gp_2.0x | CLE | 0.5909 | 0.4762 | 0.5417 | 0.5263 | 0.5652 | 22 |
|  | cwgan_gp_2.0x | PSE | 0.3636 | 0.75 | 0.3333 | 0.7742 | 0.3478 | 11 |
|  | aug_0.5x | Normal | 0.5 | 0.7273 | 0.3571 | 0.8276 | 0.4167 | 10 |
|  | aug_0.5x | CLE | 0.5 | 0.619 | 0.5789 | 0.5417 | 0.5366 | 22 |
|  | aug_0.5x | PSE | 0.5455 | 0.875 | 0.6 | 0.8485 | 0.5714 | 11 |
|  | aug_1.0x | Normal | 0.5 | 0.7576 | 0.3846 | 0.8333 | 0.4348 | 10 |
|  | aug_1.0x | CLE | 0.0909 | 0.8571 | 0.4 | 0.4737 | 0.1481 | 22 |
|  | aug_1.0x | PSE | 0.8182 | 0.5 | 0.36 | 0.8889 | 0.5 | 11 |
|  | aug_2.0x | Normal | 0.6 | 0.6667 | 0.3529 | 0.8462 | 0.4444 | 10 |
|  | aug_2.0x | CLE | 0.3182 | 0.8095 | 0.6364 | 0.5312 | 0.4242 | 22 |
|  | aug_2.0x | PSE | 0.6364 | 0.75 | 0.4667 | 0.8571 | 0.5385 | 11 |
|  | ensemble | Normal | 0.5 | 0.697 | 0.3333 | 0.8214 | 0.4 | 10 |
|  | ensemble | CLE | 0.2727 | 0.8571 | 0.6667 | 0.5294 | 0.3871 | 22 |
|  | ensemble | PSE | 0.7273 | 0.6562 | 0.4211 | 0.875 | 0.5333 | 11 |
| **48** | baseline | Normal | 0.5333 | 0.7097 | 0.4706 | 0.7586 | 0.5 | 15 |
|  | baseline | CLE | 0.5238 | 0.76 | 0.6471 | 0.6552 | 0.5789 | 21 |
|  | baseline | PSE | 0.6 | 0.8333 | 0.5 | 0.8824 | 0.5455 | 10 |
|  | cvae_0.5x | Normal | 0.2667 | 0.7419 | 0.3333 | 0.6765 | 0.2963 | 15 |
|  | cvae_0.5x | CLE | 0.5714 | 0.6 | 0.5455 | 0.625 | 0.5581 | 21 |
|  | cvae_0.5x | PSE | 0.5 | 0.8056 | 0.4167 | 0.8529 | 0.4545 | 10 |
|  | cvae_1.0x | Normal | 0.2667 | 0.8065 | 0.4 | 0.6944 | 0.32 | 15 |
|  | cvae_1.0x | CLE | 0.4762 | 0.48 | 0.4348 | 0.5217 | 0.4545 | 21 |
|  | cvae_1.0x | PSE | 0.6 | 0.8056 | 0.4615 | 0.8788 | 0.5217 | 10 |
|  | cvae_2.0x | Normal | 0.3333 | 0.8387 | 0.5 | 0.7222 | 0.4 | 15 |
|  | cvae_2.0x | CLE | 0.5714 | 0.64 | 0.5714 | 0.64 | 0.5714 | 21 |
|  | cvae_2.0x | PSE | 0.7 | 0.7778 | 0.4667 | 0.9032 | 0.56 | 10 |
|  | csdvae_0.5x | Normal | 0.4667 | 0.6452 | 0.3889 | 0.7143 | 0.4242 | 15 |
|  | csdvae_0.5x | CLE | 0.381 | 0.72 | 0.5333 | 0.5806 | 0.4444 | 21 |
|  | csdvae_0.5x | PSE | 0.5 | 0.7778 | 0.3846 | 0.8485 | 0.4348 | 10 |
|  | csdvae_1.0x | Normal | 0.4 | 0.8065 | 0.5 | 0.7353 | 0.4444 | 15 |
|  | csdvae_1.0x | CLE | 0.5238 | 0.6 | 0.5238 | 0.6 | 0.5238 | 21 |
|  | csdvae_1.0x | PSE | 0.5 | 0.7778 | 0.3846 | 0.8485 | 0.4348 | 10 |
|  | csdvae_2.0x | Normal | 0.4 | 0.7742 | 0.4615 | 0.7273 | 0.4286 | 15 |
|  | csdvae_2.0x | CLE | 0.4286 | 0.68 | 0.5294 | 0.5862 | 0.4737 | 21 |
|  | csdvae_2.0x | PSE | 0.7 | 0.75 | 0.4375 | 0.9 | 0.5385 | 10 |
|  | cwgan_gp_0.5x | Normal | 0.4667 | 0.6774 | 0.4118 | 0.7241 | 0.4375 | 15 |
|  | cwgan_gp_0.5x | CLE | 0.3333 | 0.8 | 0.5833 | 0.5882 | 0.4242 | 21 |
|  | cwgan_gp_0.5x | PSE | 0.7 | 0.7222 | 0.4118 | 0.8966 | 0.5185 | 10 |
|  | cwgan_gp_1.0x | Normal | 0.3333 | 0.6774 | 0.3333 | 0.6774 | 0.3333 | 15 |
|  | cwgan_gp_1.0x | CLE | 0.5238 | 0.64 | 0.55 | 0.6154 | 0.5366 | 21 |
|  | cwgan_gp_1.0x | PSE | 0.5 | 0.8333 | 0.4545 | 0.8571 | 0.4762 | 10 |
|  | cwgan_gp_2.0x | Normal | 0.4667 | 0.6774 | 0.4118 | 0.7241 | 0.4375 | 15 |
|  | cwgan_gp_2.0x | CLE | 0.3333 | 0.76 | 0.5385 | 0.5758 | 0.4118 | 21 |
|  | cwgan_gp_2.0x | PSE | 0.7 | 0.75 | 0.4375 | 0.9 | 0.5385 | 10 |
|  | aug_0.5x | Normal | 0.4 | 0.7742 | 0.4615 | 0.7273 | 0.4286 | 15 |
|  | aug_0.5x | CLE | 0.4286 | 0.72 | 0.5625 | 0.6 | 0.4865 | 21 |
|  | aug_0.5x | PSE | 0.8 | 0.75 | 0.4706 | 0.931 | 0.5926 | 10 |
|  | aug_1.0x | Normal | 0.4667 | 0.6452 | 0.3889 | 0.7143 | 0.4242 | 15 |
|  | aug_1.0x | CLE | 0.1905 | 0.76 | 0.4 | 0.5278 | 0.2581 | 21 |
|  | aug_1.0x | PSE | 0.7 | 0.6944 | 0.3889 | 0.8929 | 0.5 | 10 |
|  | aug_2.0x | Normal | 0.6 | 0.6129 | 0.4286 | 0.76 | 0.5 | 15 |
|  | aug_2.0x | CLE | 0.2857 | 0.92 | 0.75 | 0.6053 | 0.4138 | 21 |
|  | aug_2.0x | PSE | 0.8 | 0.75 | 0.4706 | 0.931 | 0.5926 | 10 |
|  | ensemble | Normal | 0.2667 | 0.7742 | 0.3636 | 0.6857 | 0.3077 | 15 |
|  | ensemble | CLE | 0.4286 | 0.52 | 0.4286 | 0.52 | 0.4286 | 21 |
|  | ensemble | PSE | 0.6 | 0.7778 | 0.4286 | 0.875 | 0.5 | 10 |
| **49** | baseline | Normal | 0.6 | 0.881 | 0.5455 | 0.9024 | 0.5714 | 10 |
|  | baseline | CLE | 0.4167 | 0.775 | 0.3571 | 0.8158 | 0.3846 | 12 |
|  | baseline | PSE | 0.7 | 0.7273 | 0.7778 | 0.64 | 0.7368 | 30 |
|  | cvae_0.5x | Normal | 0.9 | 0.7381 | 0.45 | 0.9688 | 0.6 | 10 |
|  | cvae_0.5x | CLE | 0.5 | 0.725 | 0.3529 | 0.8286 | 0.4138 | 12 |
|  | cvae_0.5x | PSE | 0.4667 | 0.9545 | 0.9333 | 0.5676 | 0.6222 | 30 |
|  | cvae_1.0x | Normal | 0.9 | 0.6667 | 0.3913 | 0.9655 | 0.5455 | 10 |
|  | cvae_1.0x | CLE | 0.25 | 0.725 | 0.2143 | 0.7632 | 0.2308 | 12 |
|  | cvae_1.0x | PSE | 0.4 | 0.8636 | 0.8 | 0.5135 | 0.5333 | 30 |
|  | cvae_2.0x | Normal | 0.8 | 0.6905 | 0.381 | 0.9355 | 0.5161 | 10 |
|  | cvae_2.0x | CLE | 0.5 | 0.65 | 0.3 | 0.8125 | 0.375 | 12 |
|  | cvae_2.0x | PSE | 0.3 | 0.9091 | 0.8182 | 0.4878 | 0.439 | 30 |
|  | csdvae_0.5x | Normal | 0.7 | 0.7381 | 0.3889 | 0.9118 | 0.5 | 10 |
|  | csdvae_0.5x | CLE | 0.5833 | 0.75 | 0.4118 | 0.8571 | 0.4828 | 12 |
|  | csdvae_0.5x | PSE | 0.5 | 0.9091 | 0.8824 | 0.5714 | 0.6383 | 30 |
|  | csdvae_1.0x | Normal | 0.6 | 0.7857 | 0.4 | 0.8919 | 0.48 | 10 |
|  | csdvae_1.0x | CLE | 0.6667 | 0.525 | 0.2963 | 0.84 | 0.4103 | 12 |
|  | csdvae_1.0x | PSE | 0.2667 | 0.9091 | 0.8 | 0.4762 | 0.4 | 30 |
|  | csdvae_2.0x | Normal | 0.6 | 0.7143 | 0.3333 | 0.8824 | 0.4286 | 10 |
|  | csdvae_2.0x | CLE | 0.4167 | 0.675 | 0.2778 | 0.7941 | 0.3333 | 12 |
|  | csdvae_2.0x | PSE | 0.4 | 0.8182 | 0.75 | 0.5 | 0.5217 | 30 |
|  | cwgan_gp_0.5x | Normal | 0.9 | 0.5714 | 0.3333 | 0.96 | 0.4865 | 10 |
|  | cwgan_gp_0.5x | CLE | 0.5 | 0.775 | 0.4 | 0.8378 | 0.4444 | 12 |
|  | cwgan_gp_0.5x | PSE | 0.3333 | 1 | 1 | 0.5238 | 0.5 | 30 |
|  | cwgan_gp_1.0x | Normal | 0.7 | 0.7381 | 0.3889 | 0.9118 | 0.5 | 10 |
|  | cwgan_gp_1.0x | CLE | 0.6667 | 0.7 | 0.4 | 0.875 | 0.5 | 12 |
|  | cwgan_gp_1.0x | PSE | 0.4333 | 0.9545 | 0.9286 | 0.5526 | 0.5909 | 30 |
|  | cwgan_gp_2.0x | Normal | 1 | 0.8095 | 0.5556 | 1 | 0.7143 | 10 |
|  | cwgan_gp_2.0x | CLE | 0.5833 | 0.65 | 0.3333 | 0.8387 | 0.4242 | 12 |
|  | cwgan_gp_2.0x | PSE | 0.4 | 0.9545 | 0.9231 | 0.5385 | 0.5581 | 30 |
|  | aug_0.5x | Normal | 0.9 | 0.7381 | 0.45 | 0.9688 | 0.6 | 10 |
|  | aug_0.5x | CLE | 0.4167 | 0.65 | 0.2632 | 0.7879 | 0.3226 | 12 |
|  | aug_0.5x | PSE | 0.4 | 0.9545 | 0.9231 | 0.5385 | 0.5581 | 30 |
|  | aug_1.0x | Normal | 0.8 | 0.8571 | 0.5714 | 0.9474 | 0.6667 | 10 |
|  | aug_1.0x | CLE | 0.6667 | 0.5 | 0.2857 | 0.8333 | 0.4 | 12 |
|  | aug_1.0x | PSE | 0.3 | 0.9545 | 0.9 | 0.5 | 0.45 | 30 |
|  | aug_2.0x | Normal | 0.8 | 0.6905 | 0.381 | 0.9355 | 0.5161 | 10 |
|  | aug_2.0x | CLE | 0.5833 | 0.7 | 0.3684 | 0.8485 | 0.4516 | 12 |
|  | aug_2.0x | PSE | 0.4 | 1 | 1 | 0.55 | 0.5714 | 30 |
|  | ensemble | Normal | 1 | 0.7381 | 0.4762 | 1 | 0.6452 | 10 |
|  | ensemble | CLE | 0.5833 | 0.7 | 0.3684 | 0.8485 | 0.4516 | 12 |
|  | ensemble | PSE | 0.3667 | 0.9545 | 0.9167 | 0.525 | 0.5238 | 30 |
| **50** | baseline | Normal | 0.3889 | 0.6829 | 0.35 | 0.7179 | 0.3684 | 18 |
|  | baseline | CLE | 0.1613 | 0.9286 | 0.7143 | 0.5 | 0.2632 | 31 |
|  | baseline | PSE | 1 | 0.551 | 0.3125 | 1 | 0.4762 | 10 |
|  | cvae_0.5x | Normal | 0.5 | 0.6341 | 0.375 | 0.7429 | 0.4286 | 18 |
|  | cvae_0.5x | CLE | 0.0968 | 0.8571 | 0.4286 | 0.4615 | 0.1579 | 31 |
|  | cvae_0.5x | PSE | 0.8 | 0.5918 | 0.2857 | 0.9355 | 0.4211 | 10 |
|  | cvae_1.0x | Normal | 0.4444 | 0.7073 | 0.4 | 0.7436 | 0.4211 | 18 |
|  | cvae_1.0x | CLE | 0 | 1 | 0 | 0.4746 | 0 | 31 |
|  | cvae_1.0x | PSE | 1 | 0.4082 | 0.2564 | 1 | 0.4082 | 10 |
|  | cvae_2.0x | Normal | 0.5 | 0.6585 | 0.3913 | 0.75 | 0.439 | 18 |
|  | cvae_2.0x | CLE | 0.129 | 0.9643 | 0.8 | 0.5 | 0.2222 | 31 |
|  | cvae_2.0x | PSE | 0.8 | 0.5306 | 0.2581 | 0.9286 | 0.3902 | 10 |
|  | csdvae_0.5x | Normal | 0.3889 | 0.7073 | 0.3684 | 0.725 | 0.3784 | 18 |
|  | csdvae_0.5x | CLE | 0.1613 | 0.8929 | 0.625 | 0.4902 | 0.2564 | 31 |
|  | csdvae_0.5x | PSE | 0.9 | 0.5306 | 0.2812 | 0.963 | 0.4286 | 10 |
|  | csdvae_1.0x | Normal | 0.4444 | 0.6585 | 0.3636 | 0.7297 | 0.4 | 18 |
|  | csdvae_1.0x | CLE | 0.129 | 0.9286 | 0.6667 | 0.4906 | 0.2162 | 31 |
|  | csdvae_1.0x | PSE | 0.8 | 0.5306 | 0.2581 | 0.9286 | 0.3902 | 10 |
|  | csdvae_2.0x | Normal | 0.5556 | 0.6585 | 0.4167 | 0.7714 | 0.4762 | 18 |
|  | csdvae_2.0x | CLE | 0.129 | 0.9286 | 0.6667 | 0.4906 | 0.2162 | 31 |
|  | csdvae_2.0x | PSE | 0.8 | 0.5714 | 0.2759 | 0.9333 | 0.4103 | 10 |
|  | cwgan_gp_0.5x | Normal | 0.5556 | 0.4878 | 0.3226 | 0.7143 | 0.4082 | 18 |
|  | cwgan_gp_0.5x | CLE | 0 | 0.9286 | 0 | 0.4561 | 0 | 31 |
|  | cwgan_gp_0.5x | PSE | 0.9 | 0.6531 | 0.3462 | 0.9697 | 0.5 | 10 |
|  | cwgan_gp_1.0x | Normal | 0.5 | 0.7317 | 0.45 | 0.7692 | 0.4737 | 18 |
|  | cwgan_gp_1.0x | CLE | 0.1935 | 0.9286 | 0.75 | 0.5098 | 0.3077 | 31 |
|  | cwgan_gp_1.0x | PSE | 1 | 0.5714 | 0.3226 | 1 | 0.4878 | 10 |
|  | cwgan_gp_2.0x | Normal | 0.6111 | 0.5854 | 0.3929 | 0.7742 | 0.4783 | 18 |
|  | cwgan_gp_2.0x | CLE | 0.0323 | 0.9643 | 0.5 | 0.4737 | 0.0606 | 31 |
|  | cwgan_gp_2.0x | PSE | 1 | 0.6122 | 0.3448 | 1 | 0.5128 | 10 |
|  | aug_0.5x | Normal | 0.3889 | 0.6829 | 0.35 | 0.7179 | 0.3684 | 18 |
|  | aug_0.5x | CLE | 0.1613 | 0.8571 | 0.5556 | 0.48 | 0.25 | 31 |
|  | aug_0.5x | PSE | 0.9 | 0.5714 | 0.3 | 0.9655 | 0.45 | 10 |
|  | aug_1.0x | Normal | 0.5 | 0.5854 | 0.3462 | 0.7273 | 0.4091 | 18 |
|  | aug_1.0x | CLE | 0.0968 | 1 | 1 | 0.5 | 0.1765 | 31 |
|  | aug_1.0x | PSE | 1 | 0.5918 | 0.3333 | 1 | 0.5 | 10 |
|  | aug_2.0x | Normal | 0.5 | 0.6098 | 0.36 | 0.7353 | 0.4186 | 18 |
|  | aug_2.0x | CLE | 0.0323 | 1 | 1 | 0.4828 | 0.0625 | 31 |
|  | aug_2.0x | PSE | 0.9 | 0.5102 | 0.2727 | 0.9615 | 0.4186 | 10 |
|  | ensemble | Normal | 0.4444 | 0.6829 | 0.381 | 0.7368 | 0.4103 | 18 |
|  | ensemble | CLE | 0.0968 | 1 | 1 | 0.5 | 0.1765 | 31 |
|  | ensemble | PSE | 1 | 0.4898 | 0.2857 | 1 | 0.4444 | 10 |
| **51** | baseline | Normal | 0.6923 | 0.5926 | 0.6207 | 0.6667 | 0.6545 | 26 |
|  | baseline | CLE | 0.0909 | 0.871 | 0.3333 | 0.5745 | 0.1429 | 22 |
|  | baseline | PSE | 1 | 0.7292 | 0.2778 | 1 | 0.4348 | 5 |
|  | cvae_0.5x | Normal | 0.7308 | 0.6296 | 0.6552 | 0.7083 | 0.6909 | 26 |
|  | cvae_0.5x | CLE | 0.2727 | 0.9032 | 0.6667 | 0.6364 | 0.3871 | 22 |
|  | cvae_0.5x | PSE | 1 | 0.7917 | 0.3333 | 1 | 0.5 | 5 |
|  | cvae_1.0x | Normal | 0.6923 | 0.5185 | 0.5806 | 0.6364 | 0.6316 | 26 |
|  | cvae_1.0x | CLE | 0.1818 | 0.9032 | 0.5714 | 0.6087 | 0.2759 | 22 |
|  | cvae_1.0x | PSE | 1 | 0.7917 | 0.3333 | 1 | 0.5 | 5 |
|  | cvae_2.0x | Normal | 0.7692 | 0.4815 | 0.5882 | 0.6842 | 0.6667 | 26 |
|  | cvae_2.0x | CLE | 0.1364 | 0.9355 | 0.6 | 0.6042 | 0.2222 | 22 |
|  | cvae_2.0x | PSE | 0.8 | 0.7917 | 0.2857 | 0.9744 | 0.4211 | 5 |
|  | csdvae_0.5x | Normal | 0.6538 | 0.5556 | 0.5862 | 0.625 | 0.6182 | 26 |
|  | csdvae_0.5x | CLE | 0.1818 | 0.871 | 0.5 | 0.6 | 0.2667 | 22 |
|  | csdvae_0.5x | PSE | 1 | 0.7708 | 0.3125 | 1 | 0.4762 | 5 |
|  | csdvae_1.0x | Normal | 0.6538 | 0.7778 | 0.7391 | 0.7 | 0.6939 | 26 |
|  | csdvae_1.0x | CLE | 0.3636 | 0.9032 | 0.7273 | 0.6667 | 0.4848 | 22 |
|  | csdvae_1.0x | PSE | 0.8 | 0.6875 | 0.2105 | 0.9706 | 0.3333 | 5 |
|  | csdvae_2.0x | Normal | 0.7308 | 0.5926 | 0.6333 | 0.6957 | 0.6786 | 26 |
|  | csdvae_2.0x | CLE | 0.1818 | 0.871 | 0.5 | 0.6 | 0.2667 | 22 |
|  | csdvae_2.0x | PSE | 0.8 | 0.7708 | 0.2667 | 0.9737 | 0.4 | 5 |
|  | cwgan_gp_0.5x | Normal | 0.6923 | 0.6667 | 0.6667 | 0.6923 | 0.6792 | 26 |
|  | cwgan_gp_0.5x | CLE | 0.1818 | 0.9355 | 0.6667 | 0.617 | 0.2857 | 22 |
|  | cwgan_gp_0.5x | PSE | 1 | 0.6875 | 0.25 | 1 | 0.4 | 5 |
|  | cwgan_gp_1.0x | Normal | 0.7308 | 0.5926 | 0.6333 | 0.6957 | 0.6786 | 26 |
|  | cwgan_gp_1.0x | CLE | 0.1818 | 0.871 | 0.5 | 0.6 | 0.2667 | 22 |
|  | cwgan_gp_1.0x | PSE | 1 | 0.7917 | 0.3333 | 1 | 0.5 | 5 |
|  | cwgan_gp_2.0x | Normal | 0.7308 | 0.4074 | 0.5429 | 0.6111 | 0.623 | 26 |
|  | cwgan_gp_2.0x | CLE | 0.0909 | 0.9032 | 0.4 | 0.5833 | 0.1481 | 22 |
|  | cwgan_gp_2.0x | PSE | 1 | 0.8333 | 0.3846 | 1 | 0.5556 | 5 |
|  | aug_0.5x | Normal | 0.6538 | 0.7037 | 0.68 | 0.6786 | 0.6667 | 26 |
|  | aug_0.5x | CLE | 0.1818 | 0.9032 | 0.5714 | 0.6087 | 0.2759 | 22 |
|  | aug_0.5x | PSE | 1 | 0.6667 | 0.2381 | 1 | 0.3846 | 5 |
|  | aug_1.0x | Normal | 0.6538 | 0.3704 | 0.5 | 0.5263 | 0.5667 | 26 |
|  | aug_1.0x | CLE | 0.0909 | 0.7742 | 0.2222 | 0.5455 | 0.129 | 22 |
|  | aug_1.0x | PSE | 0.8 | 0.875 | 0.4 | 0.9767 | 0.5333 | 5 |
|  | aug_2.0x | Normal | 0.5 | 0.5926 | 0.5417 | 0.5517 | 0.52 | 26 |
|  | aug_2.0x | CLE | 0.3182 | 0.6774 | 0.4118 | 0.5833 | 0.359 | 22 |
|  | aug_2.0x | PSE | 0.8 | 0.8333 | 0.3333 | 0.9756 | 0.4706 | 5 |
|  | ensemble | Normal | 0.7308 | 0.5556 | 0.6129 | 0.6818 | 0.6667 | 26 |
|  | ensemble | CLE | 0.1818 | 0.9355 | 0.6667 | 0.617 | 0.2857 | 22 |
|  | ensemble | PSE | 1 | 0.7708 | 0.3125 | 1 | 0.4762 | 5 |
| **52** | baseline | Normal | 0.4545 | 0.7317 | 0.3125 | 0.8333 | 0.3704 | 11 |
|  | baseline | CLE | 0.2105 | 0.7879 | 0.3636 | 0.6341 | 0.2667 | 19 |
|  | baseline | PSE | 0.6364 | 0.6333 | 0.56 | 0.7037 | 0.5957 | 22 |
|  | cvae_0.5x | Normal | 0.5455 | 0.7805 | 0.4 | 0.8649 | 0.4615 | 11 |
|  | cvae_0.5x | CLE | 0.4737 | 0.7879 | 0.5625 | 0.7222 | 0.5143 | 19 |
|  | cvae_0.5x | PSE | 0.6364 | 0.7667 | 0.6667 | 0.7419 | 0.6512 | 22 |
|  | cvae_1.0x | Normal | 0.1818 | 0.8537 | 0.25 | 0.7955 | 0.2105 | 11 |
|  | cvae_1.0x | CLE | 0.3158 | 0.9091 | 0.6667 | 0.6977 | 0.4286 | 19 |
|  | cvae_1.0x | PSE | 0.8636 | 0.4667 | 0.5429 | 0.8235 | 0.6667 | 22 |
|  | cvae_2.0x | Normal | 0.4545 | 0.7317 | 0.3125 | 0.8333 | 0.3704 | 11 |
|  | cvae_2.0x | CLE | 0.2105 | 0.6667 | 0.2667 | 0.5946 | 0.2353 | 19 |
|  | cvae_2.0x | PSE | 0.5909 | 0.7333 | 0.619 | 0.7097 | 0.6047 | 22 |
|  | csdvae_0.5x | Normal | 0.2727 | 0.8049 | 0.2727 | 0.8049 | 0.2727 | 11 |
|  | csdvae_0.5x | CLE | 0.5263 | 0.7576 | 0.5556 | 0.7353 | 0.5405 | 19 |
|  | csdvae_0.5x | PSE | 0.6818 | 0.7333 | 0.6522 | 0.7586 | 0.6667 | 22 |
|  | csdvae_1.0x | Normal | 0.3636 | 0.8049 | 0.3333 | 0.825 | 0.3478 | 11 |
|  | csdvae_1.0x | CLE | 0.4737 | 0.5758 | 0.3913 | 0.6552 | 0.4286 | 19 |
|  | csdvae_1.0x | PSE | 0.4091 | 0.7333 | 0.5294 | 0.6286 | 0.4615 | 22 |
|  | csdvae_2.0x | Normal | 0.6364 | 0.6098 | 0.3043 | 0.8621 | 0.4118 | 11 |
|  | csdvae_2.0x | CLE | 0.4737 | 0.8182 | 0.6 | 0.7297 | 0.5294 | 19 |
|  | csdvae_2.0x | PSE | 0.4545 | 0.8667 | 0.7143 | 0.6842 | 0.5556 | 22 |
|  | cwgan_gp_0.5x | Normal | 0.6364 | 0.8537 | 0.5385 | 0.8974 | 0.5833 | 11 |
|  | cwgan_gp_0.5x | CLE | 0.3684 | 0.9091 | 0.7 | 0.7143 | 0.4828 | 19 |
|  | cwgan_gp_0.5x | PSE | 0.8182 | 0.6333 | 0.6207 | 0.8261 | 0.7059 | 22 |
|  | cwgan_gp_1.0x | Normal | 0.2727 | 0.7561 | 0.2308 | 0.7949 | 0.25 | 11 |
|  | cwgan_gp_1.0x | CLE | 0.3684 | 0.8182 | 0.5385 | 0.6923 | 0.4375 | 19 |
|  | cwgan_gp_1.0x | PSE | 0.6818 | 0.6333 | 0.5769 | 0.7308 | 0.625 | 22 |
|  | cwgan_gp_2.0x | Normal | 0.4545 | 0.7073 | 0.2941 | 0.8286 | 0.3571 | 11 |
|  | cwgan_gp_2.0x | CLE | 0.3158 | 0.7879 | 0.4615 | 0.6667 | 0.375 | 19 |
|  | cwgan_gp_2.0x | PSE | 0.5909 | 0.7 | 0.5909 | 0.7 | 0.5909 | 22 |
|  | aug_0.5x | Normal | 0.3636 | 0.7561 | 0.2857 | 0.8158 | 0.32 | 11 |
|  | aug_0.5x | CLE | 0.1579 | 0.8182 | 0.3333 | 0.6279 | 0.2143 | 19 |
|  | aug_0.5x | PSE | 0.7273 | 0.5667 | 0.5517 | 0.7391 | 0.6275 | 22 |
|  | aug_1.0x | Normal | 0.4545 | 0.8049 | 0.3846 | 0.8462 | 0.4167 | 11 |
|  | aug_1.0x | CLE | 0.4211 | 0.8788 | 0.6667 | 0.725 | 0.5161 | 19 |
|  | aug_1.0x | PSE | 0.8636 | 0.7333 | 0.7037 | 0.88 | 0.7755 | 22 |
|  | aug_2.0x | Normal | 0.3636 | 0.7317 | 0.2667 | 0.8108 | 0.3077 | 11 |
|  | aug_2.0x | CLE | 0.4211 | 0.8788 | 0.6667 | 0.725 | 0.5161 | 19 |
|  | aug_2.0x | PSE | 0.7273 | 0.7 | 0.64 | 0.7778 | 0.6809 | 22 |
|  | ensemble | Normal | 0.1818 | 0.8293 | 0.2222 | 0.7907 | 0.2 | 11 |
|  | ensemble | CLE | 0.3684 | 0.8485 | 0.5833 | 0.7 | 0.4516 | 19 |
|  | ensemble | PSE | 0.8636 | 0.6 | 0.6129 | 0.8571 | 0.717 | 22 |
