# Supplementary material for: Synthetic data augmentation for CT-based emphysema subtype classification: A comparative evaluation of generative and classical approaches
Source: PLoS One. 2026 Aug 20;21(8):e0355850. doi: 10.1371/journal.pone.0355850 (PMC13492795; doi:10.1371/journal.pone.0355850)
Supplement: Supplementary file 7 [file pone.0355850.s007.docx]

**S6 Table.**

| **Seed** | **Method** | **True NT Pred NT** | **True NT Pred CLE** | **True NT Pred PSE** | **True CLE Pred NT** | **True CLE Pred CLE** | **True CLE Pred PSE** | **True PSE Pred NT** | **True PSE Pred CLE** | **True PSE Pred PSE** |
| --- | --- | --- | --- | --- | --- | --- | --- | --- | --- | --- |
| **43** | baseline | 15 | 1 | 0 | 20 | 3 | 6 | 3 | 1 | 11 |
|  | cvae_0.5x | 11 | 5 | 0 | 16 | 4 | 9 | 5 | 1 | 9 |
|  | cvae_1.0x | 10 | 6 | 0 | 12 | 7 | 10 | 5 | 0 | 10 |
|  | cvae_2.0x | 12 | 1 | 3 | 10 | 0 | 19 | 1 | 2 | 12 |
|  | csdvae_0.5x | 13 | 1 | 2 | 15 | 0 | 14 | 1 | 0 | 14 |
|  | csdvae_1.0x | 12 | 4 | 0 | 14 | 9 | 6 | 2 | 2 | 11 |
|  | csdvae_2.0x | 13 | 3 | 0 | 9 | 8 | 12 | 1 | 2 | 12 |
|  | cwgan_gp_0.5x | 11 | 4 | 1 | 16 | 7 | 6 | 2 | 1 | 12 |
|  | cwgan_gp_1.0x | 9 | 7 | 0 | 8 | 17 | 4 | 3 | 1 | 11 |
|  | cwgan_gp_2.0x | 12 | 2 | 2 | 16 | 4 | 9 | 3 | 1 | 11 |
|  | aug_0.5x | 11 | 5 | 0 | 14 | 4 | 11 | 0 | 2 | 13 |
|  | aug_1.0x | 14 | 1 | 1 | 16 | 2 | 11 | 1 | 0 | 14 |
|  | aug_2.0x | 12 | 3 | 1 | 10 | 7 | 12 | 0 | 4 | 11 |
|  | ensemble | 10 | 6 | 0 | 15 | 8 | 6 | 3 | 0 | 12 |
| **44** | baseline | 17 | 7 | 4 | 0 | 3 | 4 | 2 | 4 | 17 |
|  | cvae_0.5x | 14 | 6 | 8 | 3 | 2 | 2 | 0 | 2 | 21 |
|  | cvae_1.0x | 13 | 12 | 3 | 0 | 6 | 1 | 2 | 8 | 13 |
|  | cvae_2.0x | 14 | 8 | 6 | 1 | 4 | 2 | 3 | 4 | 16 |
|  | csdvae_0.5x | 16 | 6 | 6 | 2 | 3 | 2 | 3 | 2 | 18 |
|  | csdvae_1.0x | 9 | 14 | 5 | 2 | 3 | 2 | 2 | 2 | 19 |
|  | csdvae_2.0x | 11 | 16 | 1 | 2 | 4 | 1 | 5 | 7 | 11 |
|  | cwgan_gp_0.5x | 11 | 13 | 4 | 0 | 3 | 4 | 4 | 3 | 16 |
|  | cwgan_gp_1.0x | 16 | 8 | 4 | 1 | 4 | 2 | 3 | 4 | 16 |
|  | cwgan_gp_2.0x | 11 | 15 | 2 | 1 | 2 | 4 | 1 | 10 | 12 |
|  | aug_0.5x | 8 | 13 | 7 | 0 | 5 | 2 | 3 | 2 | 18 |
|  | aug_1.0x | 14 | 8 | 6 | 1 | 2 | 4 | 0 | 2 | 21 |
|  | aug_2.0x | 14 | 6 | 8 | 1 | 2 | 4 | 1 | 5 | 17 |
|  | ensemble | 14 | 10 | 4 | 0 | 5 | 2 | 1 | 2 | 20 |
| **45** | baseline | 6 | 4 | 11 | 5 | 8 | 19 | 0 | 0 | 7 |
|  | cvae_0.5x | 6 | 3 | 12 | 6 | 3 | 23 | 0 | 0 | 7 |
|  | cvae_1.0x | 9 | 1 | 11 | 12 | 5 | 15 | 0 | 0 | 7 |
|  | cvae_2.0x | 10 | 4 | 7 | 6 | 7 | 19 | 0 | 0 | 7 |
|  | csdvae_0.5x | 8 | 2 | 11 | 9 | 3 | 20 | 0 | 0 | 7 |
|  | csdvae_1.0x | 10 | 4 | 7 | 8 | 3 | 21 | 0 | 0 | 7 |
|  | csdvae_2.0x | 9 | 0 | 12 | 7 | 3 | 22 | 0 | 0 | 7 |
|  | cwgan_gp_0.5x | 10 | 3 | 8 | 9 | 4 | 19 | 0 | 0 | 7 |
|  | cwgan_gp_1.0x | 9 | 1 | 11 | 8 | 3 | 21 | 0 | 0 | 7 |
|  | cwgan_gp_2.0x | 7 | 0 | 14 | 11 | 0 | 21 | 0 | 0 | 7 |
|  | aug_0.5x | 11 | 2 | 8 | 10 | 0 | 22 | 0 | 0 | 7 |
|  | aug_1.0x | 10 | 1 | 10 | 13 | 0 | 19 | 0 | 0 | 7 |
|  | aug_2.0x | 12 | 2 | 7 | 13 | 3 | 16 | 0 | 0 | 7 |
|  | ensemble | 9 | 2 | 10 | 10 | 3 | 19 | 0 | 0 | 7 |
| **46** | baseline | 10 | 1 | 5 | 15 | 4 | 7 | 1 | 4 | 4 |
|  | cvae_0.5x | 9 | 1 | 6 | 14 | 4 | 8 | 0 | 4 | 5 |
|  | cvae_1.0x | 8 | 2 | 6 | 12 | 5 | 9 | 0 | 3 | 6 |
|  | cvae_2.0x | 9 | 1 | 6 | 14 | 2 | 10 | 0 | 2 | 7 |
|  | csdvae_0.5x | 7 | 1 | 8 | 15 | 5 | 6 | 0 | 2 | 7 |
|  | csdvae_1.0x | 7 | 4 | 5 | 12 | 8 | 6 | 0 | 4 | 5 |
|  | csdvae_2.0x | 9 | 2 | 5 | 13 | 6 | 7 | 0 | 6 | 3 |
|  | cwgan_gp_0.5x | 8 | 2 | 6 | 14 | 10 | 2 | 0 | 6 | 3 |
|  | cwgan_gp_1.0x | 10 | 0 | 6 | 15 | 3 | 8 | 1 | 0 | 8 |
|  | cwgan_gp_2.0x | 7 | 4 | 5 | 13 | 6 | 7 | 0 | 5 | 4 |
|  | aug_0.5x | 10 | 0 | 6 | 13 | 3 | 10 | 1 | 2 | 6 |
|  | aug_1.0x | 9 | 1 | 6 | 16 | 3 | 7 | 4 | 1 | 4 |
|  | aug_2.0x | 10 | 0 | 6 | 13 | 6 | 7 | 0 | 6 | 3 |
|  | ensemble | 9 | 1 | 6 | 13 | 5 | 8 | 1 | 3 | 5 |
| **47** | baseline | 4 | 2 | 4 | 8 | 5 | 9 | 0 | 3 | 8 |
|  | cvae_0.5x | 4 | 2 | 4 | 6 | 7 | 9 | 0 | 3 | 8 |
|  | cvae_1.0x | 6 | 1 | 3 | 11 | 5 | 6 | 2 | 2 | 7 |
|  | cvae_2.0x | 6 | 1 | 3 | 8 | 6 | 8 | 1 | 6 | 4 |
|  | csdvae_0.5x | 5 | 2 | 3 | 6 | 8 | 8 | 1 | 4 | 6 |
|  | csdvae_1.0x | 5 | 1 | 4 | 9 | 7 | 6 | 1 | 3 | 7 |
|  | csdvae_2.0x | 6 | 1 | 3 | 7 | 9 | 6 | 2 | 3 | 6 |
|  | cwgan_gp_0.5x | 3 | 3 | 4 | 7 | 8 | 7 | 1 | 3 | 7 |
|  | cwgan_gp_1.0x | 4 | 2 | 4 | 7 | 12 | 3 | 2 | 3 | 6 |
|  | cwgan_gp_2.0x | 3 | 4 | 3 | 4 | 13 | 5 | 0 | 7 | 4 |
|  | aug_0.5x | 5 | 3 | 2 | 9 | 11 | 2 | 0 | 5 | 6 |
|  | aug_1.0x | 5 | 2 | 3 | 7 | 2 | 13 | 1 | 1 | 9 |
|  | aug_2.0x | 6 | 0 | 4 | 11 | 7 | 4 | 0 | 4 | 7 |
|  | ensemble | 5 | 1 | 4 | 9 | 6 | 7 | 1 | 2 | 8 |
| **48** | baseline | 8 | 4 | 3 | 7 | 11 | 3 | 2 | 2 | 6 |
|  | cvae_0.5x | 4 | 8 | 3 | 5 | 12 | 4 | 3 | 2 | 5 |
|  | cvae_1.0x | 4 | 9 | 2 | 6 | 10 | 5 | 0 | 4 | 6 |
|  | cvae_2.0x | 5 | 7 | 3 | 4 | 12 | 5 | 1 | 2 | 7 |
|  | csdvae_0.5x | 7 | 5 | 3 | 8 | 8 | 5 | 3 | 2 | 5 |
|  | csdvae_1.0x | 6 | 7 | 2 | 4 | 11 | 6 | 2 | 3 | 5 |
|  | csdvae_2.0x | 6 | 5 | 4 | 7 | 9 | 5 | 0 | 3 | 7 |
|  | cwgan_gp_0.5x | 7 | 4 | 4 | 8 | 7 | 6 | 2 | 1 | 7 |
|  | cwgan_gp_1.0x | 5 | 6 | 4 | 8 | 11 | 2 | 2 | 3 | 5 |
|  | cwgan_gp_2.0x | 7 | 4 | 4 | 9 | 7 | 5 | 1 | 2 | 7 |
|  | aug_0.5x | 6 | 6 | 3 | 6 | 9 | 6 | 1 | 1 | 8 |
|  | aug_1.0x | 7 | 3 | 5 | 11 | 4 | 6 | 0 | 3 | 7 |
|  | aug_2.0x | 9 | 1 | 5 | 11 | 6 | 4 | 1 | 1 | 8 |
|  | ensemble | 4 | 9 | 2 | 6 | 9 | 6 | 1 | 3 | 6 |
| **49** | baseline | 6 | 0 | 4 | 5 | 5 | 2 | 0 | 9 | 21 |
|  | cvae_0.5x | 9 | 1 | 0 | 5 | 6 | 1 | 6 | 10 | 14 |
|  | cvae_1.0x | 9 | 1 | 0 | 6 | 3 | 3 | 8 | 10 | 12 |
|  | cvae_2.0x | 8 | 2 | 0 | 4 | 6 | 2 | 9 | 12 | 9 |
|  | csdvae_0.5x | 7 | 1 | 2 | 5 | 7 | 0 | 6 | 9 | 15 |
|  | csdvae_1.0x | 6 | 3 | 1 | 3 | 8 | 1 | 6 | 16 | 8 |
|  | csdvae_2.0x | 6 | 3 | 1 | 4 | 5 | 3 | 8 | 10 | 12 |
|  | cwgan_gp_0.5x | 9 | 1 | 0 | 6 | 6 | 0 | 12 | 8 | 10 |
|  | cwgan_gp_1.0x | 7 | 2 | 1 | 4 | 8 | 0 | 7 | 10 | 13 |
|  | cwgan_gp_2.0x | 10 | 0 | 0 | 4 | 7 | 1 | 4 | 14 | 12 |
|  | aug_0.5x | 9 | 0 | 1 | 7 | 5 | 0 | 4 | 14 | 12 |
|  | aug_1.0x | 8 | 2 | 0 | 3 | 8 | 1 | 3 | 18 | 9 |
|  | aug_2.0x | 8 | 2 | 0 | 5 | 7 | 0 | 8 | 10 | 12 |
|  | ensemble | 10 | 0 | 0 | 4 | 7 | 1 | 7 | 12 | 11 |
| **50** | baseline | 7 | 2 | 9 | 13 | 5 | 13 | 0 | 0 | 10 |
|  | cvae_0.5x | 9 | 2 | 7 | 15 | 3 | 13 | 0 | 2 | 8 |
|  | cvae_1.0x | 8 | 0 | 10 | 12 | 0 | 19 | 0 | 0 | 10 |
|  | cvae_2.0x | 9 | 1 | 8 | 12 | 4 | 15 | 2 | 0 | 8 |
|  | csdvae_0.5x | 7 | 2 | 9 | 12 | 5 | 14 | 0 | 1 | 9 |
|  | csdvae_1.0x | 8 | 0 | 10 | 14 | 4 | 13 | 0 | 2 | 8 |
|  | csdvae_2.0x | 10 | 0 | 8 | 14 | 4 | 13 | 0 | 2 | 8 |
|  | cwgan_gp_0.5x | 10 | 2 | 6 | 20 | 0 | 11 | 1 | 0 | 9 |
|  | cwgan_gp_1.0x | 9 | 2 | 7 | 11 | 6 | 14 | 0 | 0 | 10 |
|  | cwgan_gp_2.0x | 11 | 1 | 6 | 17 | 1 | 13 | 0 | 0 | 10 |
|  | aug_0.5x | 7 | 3 | 8 | 13 | 5 | 13 | 0 | 1 | 9 |
|  | aug_1.0x | 9 | 0 | 9 | 17 | 3 | 11 | 0 | 0 | 10 |
|  | aug_2.0x | 9 | 0 | 9 | 15 | 1 | 15 | 1 | 0 | 9 |
|  | ensemble | 8 | 0 | 10 | 13 | 3 | 15 | 0 | 0 | 10 |
| **51** | baseline | 18 | 4 | 4 | 11 | 2 | 9 | 0 | 0 | 5 |
|  | cvae_0.5x | 19 | 3 | 4 | 10 | 6 | 6 | 0 | 0 | 5 |
|  | cvae_1.0x | 18 | 3 | 5 | 13 | 4 | 5 | 0 | 0 | 5 |
|  | cvae_2.0x | 20 | 2 | 4 | 13 | 3 | 6 | 1 | 0 | 4 |
|  | csdvae_0.5x | 17 | 4 | 5 | 12 | 4 | 6 | 0 | 0 | 5 |
|  | csdvae_1.0x | 17 | 3 | 6 | 5 | 8 | 9 | 1 | 0 | 4 |
|  | csdvae_2.0x | 19 | 4 | 3 | 10 | 4 | 8 | 1 | 0 | 4 |
|  | cwgan_gp_0.5x | 18 | 2 | 6 | 9 | 4 | 9 | 0 | 0 | 5 |
|  | cwgan_gp_1.0x | 19 | 4 | 3 | 11 | 4 | 7 | 0 | 0 | 5 |
|  | cwgan_gp_2.0x | 19 | 3 | 4 | 16 | 2 | 4 | 0 | 0 | 5 |
|  | aug_0.5x | 17 | 3 | 6 | 8 | 4 | 10 | 0 | 0 | 5 |
|  | aug_1.0x | 17 | 7 | 2 | 16 | 2 | 4 | 1 | 0 | 4 |
|  | aug_2.0x | 13 | 9 | 4 | 11 | 7 | 4 | 0 | 1 | 4 |
|  | ensemble | 19 | 2 | 5 | 12 | 4 | 6 | 0 | 0 | 5 |
| **52** | baseline | 5 | 1 | 5 | 9 | 4 | 6 | 2 | 6 | 14 |
|  | cvae_0.5x | 6 | 3 | 2 | 5 | 9 | 5 | 4 | 4 | 14 |
|  | cvae_1.0x | 2 | 2 | 7 | 4 | 6 | 9 | 2 | 1 | 19 |
|  | cvae_2.0x | 5 | 4 | 2 | 9 | 4 | 6 | 2 | 7 | 13 |
|  | csdvae_0.5x | 3 | 3 | 5 | 6 | 10 | 3 | 2 | 5 | 15 |
|  | csdvae_1.0x | 4 | 4 | 3 | 5 | 9 | 5 | 3 | 10 | 9 |
|  | csdvae_2.0x | 7 | 2 | 2 | 8 | 9 | 2 | 8 | 4 | 10 |
|  | cwgan_gp_0.5x | 7 | 0 | 4 | 5 | 7 | 7 | 1 | 3 | 18 |
|  | cwgan_gp_1.0x | 3 | 3 | 5 | 6 | 7 | 6 | 4 | 3 | 15 |
|  | cwgan_gp_2.0x | 5 | 4 | 2 | 6 | 6 | 7 | 6 | 3 | 13 |
|  | aug_0.5x | 4 | 2 | 5 | 8 | 3 | 8 | 2 | 4 | 16 |
|  | aug_1.0x | 5 | 2 | 4 | 7 | 8 | 4 | 1 | 2 | 19 |
|  | aug_2.0x | 4 | 1 | 6 | 8 | 8 | 3 | 3 | 3 | 16 |
|  | ensemble | 2 | 4 | 5 | 5 | 7 | 7 | 2 | 1 | 19 |
