# Supplementary material for: Synthetic data augmentation for CT-based emphysema subtype classification: A comparative evaluation of generative and classical approaches
Source: PLoS One. 2026 Aug 20;21(8):e0355850. doi: 10.1371/journal.pone.0355850 (PMC13492795; doi:10.1371/journal.pone.0355850)
Supplement: Supplementary file 8 [file pone.0355850.s008.docx]

**S7 Table.**

| **Seed** | **Method** | **MMD pixel** | **MMD feature** | **Balanced Accuracy** |
| --- | --- | --- | --- | --- |
| **43** | cvae_0.5x | 0.031426 | 0.335451 | 0.4751 |
| **43** | cvae_1.0x | 0.019878 | 0.0813 | 0.511 |
| **43** | cvae_2.0x | 0.017779 | 0.089176 | 0.5167 |
| **43** | csdvae_0.5x | 0.043934 | 0.316546 | 0.5819 |
| **43** | csdvae_1.0x | 0.031222 | 0.075253 | 0.5979 |
| **43** | csdvae_2.0x | 0.02692 | 0.114956 | 0.6295 |
| **43** | cwgan_gp_0.5x | 0.012685 | 0.251761 | 0.5763 |
| **43** | cwgan_gp_1.0x | 0.006591 | 0.201062 | 0.6273 |
| **43** | cwgan_gp_2.0x | 0.007684 | 0.306036 | 0.5404 |
| **43** | aug_0.5x | 0.01446 | 0.262588 | 0.564 |
| **43** | aug_1.0x | 0.00547 | 0.098888 | 0.6258 |
| **43** | aug_2.0x | 0.004876 | 0.103536 | 0.5749 |
| **44** | cvae_0.5x | 0.029769 | 0.247725 | 0.5663 |
| **44** | cvae_1.0x | 0.034928 | 0.092359 | 0.6289 |
| **44** | cvae_2.0x | 0.022386 | 0.067468 | 0.589 |
| **44** | csdvae_0.5x | 0.033294 | 0.26765 | 0.5942 |
| **44** | csdvae_1.0x | 0.038307 | 0.092545 | 0.5254 |
| **44** | csdvae_2.0x | 0.021771 | 0.059242 | 0.4808 |
| **44** | cwgan_gp_0.5x | 0.008363 | 0.432807 | 0.5057 |
| **44** | cwgan_gp_1.0x | 0.006511 | 0.194728 | 0.6128 |
| **44** | cwgan_gp_2.0x | 0.004191 | 0.184865 | 0.4001 |
| **44** | aug_0.5x | 0.005944 | 0.235054 | 0.5942 |
| **44** | aug_1.0x | 0.011134 | 0.100823 | 0.5663 |
| **44** | aug_2.0x | 0.00683 | 0.085403 | 0.5083 |
| **45** | cvae_0.5x | 0.024673 | 0.301795 | 0.4598 |
| **45** | cvae_1.0x | 0.034275 | 0.097322 | 0.5283 |
| **45** | cvae_2.0x | 0.027042 | 0.164735 | 0.565 |
| **45** | csdvae_0.5x | 0.020286 | 0.308209 | 0.4916 |
| **45** | csdvae_1.0x | 0.025183 | 0.098379 | 0.5233 |
| **45** | csdvae_2.0x | 0.020494 | 0.148419 | 0.5074 |
| **45** | cwgan_gp_0.5x | 0.007856 | 0.391364 | 0.5337 |
| **45** | cwgan_gp_1.0x | 0.005484 | 0.098385 | 0.5074 |
| **45** | cwgan_gp_2.0x | 0.005759 | 0.234395 | 0.4444 |
| **45** | aug_0.5x | 0.004489 | 0.276492 | 0.5079 |
| **45** | aug_1.0x | 0.008498 | 0.096914 | 0.4921 |
| **45** | aug_2.0x | 0.006646 | 0.142754 | 0.5551 |
| **46** | cvae_0.5x | 0.024813 | 0.308266 | 0.424 |
| **46** | cvae_1.0x | 0.024389 | 0.119009 | 0.453 |
| **46** | cvae_2.0x | 0.024936 | 0.156673 | 0.4724 |
| **46** | csdvae_0.5x | 0.022764 | 0.258944 | 0.4692 |
| **46** | csdvae_1.0x | 0.022533 | 0.088006 | 0.4336 |
| **46** | csdvae_2.0x | 0.022816 | 0.12124 | 0.3755 |
| **46** | cwgan_gp_0.5x | 0.008625 | 0.301453 | 0.406 |
| **46** | cwgan_gp_1.0x | 0.005546 | 0.104456 | 0.5431 |
| **46** | cwgan_gp_2.0x | 0.007268 | 0.142199 | 0.3709 |
| **46** | aug_0.5x | 0.008956 | 0.180911 | 0.469 |
| **46** | aug_1.0x | 0.003962 | 0.060344 | 0.3741 |
| **46** | aug_2.0x | 0.003919 | 0.099876 | 0.3964 |
| **47** | cvae_0.5x | 0.024238 | 0.418856 | 0.4818 |
| **47** | cvae_1.0x | 0.024132 | 0.13017 | 0.4879 |
| **47** | cvae_2.0x | 0.014952 | 0.151131 | 0.4121 |
| **47** | csdvae_0.5x | 0.029479 | 0.39503 | 0.4697 |
| **47** | csdvae_1.0x | 0.029215 | 0.097834 | 0.4848 |
| **47** | csdvae_2.0x | 0.016709 | 0.14762 | 0.5182 |
| **47** | cwgan_gp_0.5x | 0.008212 | 0.354465 | 0.4333 |
| **47** | cwgan_gp_1.0x | 0.004111 | 0.103922 | 0.497 |
| **47** | cwgan_gp_2.0x | 0.008248 | 0.172442 | 0.4182 |
| **47** | aug_0.5x | 0.012035 | 0.380006 | 0.5152 |
| **47** | aug_1.0x | 0.00487 | 0.097932 | 0.4697 |
| **47** | aug_2.0x | 0.005168 | 0.105448 | 0.5182 |
| **48** | cvae_0.5x | 0.039529 | 0.293268 | 0.446 |
| **48** | cvae_1.0x | 0.021859 | 0.093933 | 0.4476 |
| **48** | cvae_2.0x | 0.015896 | 0.138845 | 0.5349 |
| **48** | csdvae_0.5x | 0.040248 | 0.25817 | 0.4492 |
| **48** | csdvae_1.0x | 0.021898 | 0.075864 | 0.4746 |
| **48** | csdvae_2.0x | 0.017169 | 0.172711 | 0.5095 |
| **48** | cwgan_gp_0.5x | 0.006193 | 0.339418 | 0.5 |
| **48** | cwgan_gp_1.0x | 0.004418 | 0.08417 | 0.4524 |
| **48** | cwgan_gp_2.0x | 0.005151 | 0.101833 | 0.5 |
| **48** | aug_0.5x | 0.007941 | 0.293595 | 0.5429 |
| **48** | aug_1.0x | 0.008078 | 0.105769 | 0.4524 |
| **48** | aug_2.0x | 0.004174 | 0.120447 | 0.5619 |
| **49** | cvae_0.5x | 0.042323 | 0.319358 | 0.6222 |
| **49** | cvae_1.0x | 0.032294 | 0.175975 | 0.5167 |
| **49** | cvae_2.0x | 0.024688 | 0.183562 | 0.5333 |
| **49** | csdvae_0.5x | 0.048116 | 0.314304 | 0.5944 |
| **49** | csdvae_1.0x | 0.037124 | 0.179433 | 0.5111 |
| **49** | csdvae_2.0x | 0.027389 | 0.201658 | 0.4722 |
| **49** | cwgan_gp_0.5x | 0.012142 | 0.269452 | 0.5778 |
| **49** | cwgan_gp_1.0x | 0.012777 | 0.278758 | 0.6 |
| **49** | cwgan_gp_2.0x | 0.015677 | 0.31921 | 0.6611 |
| **49** | aug_0.5x | 0.009621 | 0.23601 | 0.5722 |
| **49** | aug_1.0x | 0.006196 | 0.119103 | 0.5889 |
| **49** | aug_2.0x | 0.005305 | 0.079884 | 0.5944 |
| **50** | cvae_0.5x | 0.027973 | 0.529345 | 0.4656 |
| **50** | cvae_1.0x | 0.022364 | 0.121571 | 0.4815 |
| **50** | cvae_2.0x | 0.02118 | 0.139984 | 0.4763 |
| **50** | csdvae_0.5x | 0.023466 | 0.498904 | 0.4834 |
| **50** | csdvae_1.0x | 0.019046 | 0.115636 | 0.4578 |
| **50** | csdvae_2.0x | 0.018024 | 0.15373 | 0.4949 |
| **50** | cwgan_gp_0.5x | 0.012973 | 0.516982 | 0.4852 |
| **50** | cwgan_gp_1.0x | 0.004416 | 0.155062 | 0.5645 |
| **50** | cwgan_gp_2.0x | 0.004068 | 0.197767 | 0.5478 |
| **50** | aug_0.5x | 0.010201 | 0.422307 | 0.4834 |
| **50** | aug_1.0x | 0.005543 | 0.099027 | 0.5323 |
| **50** | aug_2.0x | 0.008105 | 0.069843 | 0.4774 |
| **51** | cvae_0.5x | 0.017787 | 0.243903 | 0.6678 |
| **51** | cvae_1.0x | 0.021439 | 0.082715 | 0.6247 |
| **51** | cvae_2.0x | 0.010078 | 0.133871 | 0.5685 |
| **51** | csdvae_0.5x | 0.011573 | 0.238389 | 0.6119 |
| **51** | csdvae_1.0x | 0.018076 | 0.083387 | 0.6058 |
| **51** | csdvae_2.0x | 0.006017 | 0.138483 | 0.5709 |
| **51** | cwgan_gp_0.5x | 0.008642 | 0.271852 | 0.6247 |
| **51** | cwgan_gp_1.0x | 0.007876 | 0.07875 | 0.6375 |
| **51** | cwgan_gp_2.0x | 0.009311 | 0.186668 | 0.6072 |
| **51** | aug_0.5x | 0.004139 | 0.204265 | 0.6119 |
| **51** | aug_1.0x | 0.005504 | 0.052286 | 0.5149 |
| **51** | aug_2.0x | 0.003629 | 0.080439 | 0.5394 |
| **52** | cvae_0.5x | 0.019012 | 0.236545 | 0.5518 |
| **52** | cvae_1.0x | 0.021959 | 0.123634 | 0.4537 |
| **52** | cvae_2.0x | 0.016733 | 0.216295 | 0.4187 |
| **52** | csdvae_0.5x | 0.023459 | 0.249758 | 0.4936 |
| **52** | csdvae_1.0x | 0.027433 | 0.146351 | 0.4155 |
| **52** | csdvae_2.0x | 0.02125 | 0.193624 | 0.5215 |
| **52** | cwgan_gp_0.5x | 0.010419 | 0.452566 | 0.6077 |
| **52** | cwgan_gp_1.0x | 0.006729 | 0.30533 | 0.441 |
| **52** | cwgan_gp_2.0x | 0.007457 | 0.384989 | 0.4537 |
| **52** | aug_0.5x | 0.009855 | 0.32465 | 0.4163 |
| **52** | aug_1.0x | 0.00563 | 0.141973 | 0.5797 |
| **52** | aug_2.0x | 0.01087 | 0.190148 | 0.504 |
